# RNA-binding protein MSI2 isoforms expression and regulation in progression of triple-negative breast cancer

**DOI:** 10.1186/s13046-020-01587-x

**Published:** 2020-05-24

**Authors:** Ming Li, An-qi Li, Shu-ling Zhou, Hong Lv, Ping Wei, Wen-tao Yang

**Affiliations:** 1 Department of Pathology, Fudan University Shanghai Cancer Center, Shanghai, 200032 China; 2 Department of Oncology, Shanghai Medical College, Fudan University, Shanghai, China; 3 Institute of Pathology, Fudan University, Shanghai, China; 4 Cancer Institute, Fudan University Shanghai Cancer Center, Shanghai, China

**Keywords:** Triple-negative breast cancer, MSI2, Isoforms, TP53INP1, Metastasis

## Abstract

**Background:**

The RNA-binding protein Musashi-2 (MSI2) has been implicated in the tumorigenesis and tumor progression of some human cancers. MSI2 has also been reported to suppress tumor epithelial-to-mesenchymal transition (EMT) progression in breast cancer, and low MSI2 expression is associated with poor outcomes for breast cancer patients; however, the underlying mechanisms have not been fully investigated. This study investigated the expression and phenotypic functions of two major alternatively spliced MSI2 isoforms (MSI2a and MSI2b) and the potential molecular mechanisms involved in triple-negative breast cancer (TNBC) progression.

**Methods:**

The Illumina sequencing platform was used to analyze the mRNA transcriptomes of TNBC and normal tissues, while quantitative reverse transcription-polymerase chain reaction and immunohistochemistry validated MSI2 isoform expression in breast cancer tissues. The effects of MSI2a and MSI2b on TNBC cells were assayed in vitro and in vivo. RNA immunoprecipitation (RIP) and RNA sequencing were performed to identify the potential mRNA targets of MSI2a, and RIP and luciferase analyses were used to confirm the mRNA targets of MSI2.

**Results:**

MSI2 expression in TNBC tissues was significantly downregulated compared to that in normal tissues. In TNBC, MSI2a expression was associated with poor overall survival of patients. MSI2a overexpression in vitro and in vivo inhibited TNBC cell invasion as well as extracellular signal-regulated kinase 1/2 (ERK1/2) activity. However, MSI2b overexpression had no significant effects on TNBC cell migration. Mechanistically, MSI2a expression promoted TP53INP1 mRNA stability by its interaction with the 3′-untranslated region of TP53INP1 mRNA. Furthermore, TP53INP1 knockdown reversed MSI2a-induced suppression of TNBC cell invasion, whereas ectopic expression of TP53INP1 and inhibition of ERK1/2 activity blocked MSI2 knockdown-induced TNBC cell invasion.

**Conclusions:**

The current study demonstrated that MSI2a is the predominant functional isoform of MSI2 proteins in TNBC, that its downregulation is associated with TNBC progression and poor prognosis and that MSI2a expression inhibited TNBC invasion by stabilizing TP53INP1 mRNA and inhibiting ERK1/2 activity. Overall, our study provides new insights into the isoform-specific roles of MSI2a and MSI2b in the tumor progression of TNBC, allowing for novel therapeutic strategies to be developed for TNBC.

## Background

Although there have been great advancements in the early detection, treatment options, and preventive measures for breast cancer in recent decades, breast cancer remains one of the most significant health burdens for women worldwide [1, 2]. Triple-negative breast cancer (TNBC) is characterized by the lack of estrogen receptor, progesterone receptor expression, and human epidermal growth factor receptor 2 amplification [3]. TNBC accounts for approximately 15–20% of all breast cancers and is a generally aggressive disease with high rates of recurrence and distant metastasis, partially due to the lack of effective targeted therapies [4–6]. Thus, a better understanding of TNBC biology and pathogenesis may help in the identification of novel strategies for the prevention and treatment of this cancer.

Posttranscriptional regulation controls gene expression and cellular behavior. RNA-binding proteins (RBPs) are key molecules that regulate the splicing, polyadenylation, localization, translation, and decay of target mRNAs [7]. Accumulating evidence indicates that RBP dysfunction or aberrant expression contributes to cancer initiation and progression; therefore, RBPs might serve as a good therapeutic target for cancer therapy [8].

The RBP Musashi-2 (MSI2), a member of the Musashi gene family, has been characterized as a cancer-driver gene in some tumors [9, 10]. For example, in acute myeloid leukemia (AML), MSI2 expression has been shown to be an adverse prognostic marker that maintains the self-renewal program in AML by interacting with HOXA9, MYC, and IKZF2 mRNAs [11]. In lung adenocarcinoma, MSI2 has been reported to contribute to cancer invasion by regulating TGFβR1/SMAD3 signaling and can serve as a predictive biomarker of non-small-cell lung cancer progression [12]. However, there are also some reports that MSI2 has distinct functions. MSI2 overexpression blocked MIN6 cell (a mouse insulinoma cell line) proliferation, whereas MSI2 knockdown augmented MIN6 cell proliferation [13]. A recent study of breast cancer suggested that MSI2 can also suppress epithelial-to-mesenchymal transition (EMT) progression in TNBC MDA-MB-231 cells by regulating the translation of epithelial genes [14]. Another study of luminal breast cancer demonstrated that MSI2 promotes luminal cell growth, although survival analysis indicated that high MSI2 expression is associated with favorable outcomes for breast cancer patients [15]. These divergent functions of MSI2, especially in breast cancer, suggest that it might have additional molecular mechanisms that need to be further investigated.

Mammalian MSI2 has four isoforms, all of which contain two conserved RNA-recognition motifs (RRMs) for binding to the target mRNAs but differ in the N-terminus or C-terminus. Most studies have focused on MSI2a (MSI2a), the canonical and full-length isoform, while only a few studies have focused on the functions of different MSI2 isoforms. Overexpression of MSI2a, but not of isoform b (MSI2b), enhances the self-renewal capacity of embryonic stem cells (ESCs), yet both isoforms of MSI2 are required to maintain the self-renewal of ESCs [16]. Another study demonstrated that canonical MSI2a is subject to site-specific phosphorylation at the C-terminus, converting MSI2 from a repressor to an activator of target mRNA translation; in contrast, the truncated isoform of MSI2 (isoform b) that lacks regulatory phosphorylation sites could promote cell transformation and is overexpressed in multiple cancers [17]. These findings indicate that alternately spliced isoforms of MSI2 possess distinct functional and regulatory properties and need to be further investigated.

In this study, we conducted ex vivo, in vitro, and in vivo experiments as follows: 1) transcriptome analysis to profile the expression levels of different RBPs in TNBC; 2) clarification of MSI2 isoform expression in breast cancer patients; 3) overexpression or knockdown of MSI2a and -b in TNBC cells to assess the role of MSI2 in breast cancer cells in vitro and in nude mice in vivo; 4) exploration of the underlying molecular mechanism of the anti-TNBC activity of MSI2a in vitro and in vivo; and 5) identification of MSI2a-interacting and binding partners, which mediate MSI2 antitumor activities. In particular, our study focused on the posttranscriptional regulation of gene expression by MSI2a in TNBC progression. The results provide detailed insight into the different expression patterns and functions of the MSI2 isoforms and the mechanism of MSI2a in TNBC development and progression.

## Methods

### Patients and tissue samples

In this study, we analyzed four independent cohorts of breast cancer samples from a total of 569 invasive ductal breast cancer patients who received medical care at the Department of Breast Surgery, Fudan University Shanghai Cancer Center (Shanghai, China). The clinicopathological and survival data were retrieved from the medical records of the patients. This study was approved by the Clinical Research Ethics Committee of Fudan University Shanghai Cancer Center and was conducted according to their guidelines; each participant signed an informed consent form before they were enrolled in this study. Specifically, cohort 1 contained 25 TNBC tissues and 5 adjacent normal breast tissues (ANTs) collected between January 2013 and May 2015 and were used for transcriptome profiling analysis (Table S1); cohort 2 included 27 paired TNBC tissues and ANTs (Table S1) for quantitative real-time polymerase chain reaction (qRT-PCR) analysis; cohort 3 consisted of 129 invasive ductal breast cancer tissue samples that were collected between February 2007 and October 2009 and were used for qRT-PCR analysis of MSI2a and MSI2b expression; and cohort 4 with 388 paraffin-embedded tissue blocks between September 2001 and January 2008 to construct tissue microarrays was used for immunohistochemical analysis of the MSI2a protein.

In addition, we downloaded datasets of MSI2 and TP53INP1 expression from The Cancer Genome Atlas (TCGA) (http://cancergenome.nih.gov/), Cancer Cell Line Encyclopedia (CCLE) (https://portals.broadinstitute.org/ccle_legacy/search/searchResult), and GSE76250 (https://www.ncbi.nlm.nih.gov/geo/geo2r/? acc = GSE76250). We then performed bioinformatics analysis on these data for comparison with our current data.

### RNA isolation and qRT-PCR

Fresh tissue specimens were collected and preserved in RNAlater Stabilization Solution and were then microdissected to reach at least 80% of tumor cells in the breast cancer specimens. Total RNA was then isolated from the tissues and cell lines using TRIzol reagent (Invitrogen, Carlsbad, CA, USA) and was reverse transcribed into cDNA using the PrimeScript RT Reagent Kit (TaKaRa, Dalian, China) according to the manufacturers’ protocols. The resulting cDNA samples were subjected to qPCR amplification with an ABI 7900HT qPCR instrument (Applied Biosystems) using SYBR Green (TaKaRa, Dalian, China) and primers for detection of the relative MSI2, TP53INP1, and β-actin mRNA levels, while β-actin was used as an internal control. The primer sequences were as follows: total MSI2, 5′-GGTCATGAGAGATCCCACTACG-3′ and 5′-TCTACACTTGCTGGGTCTGC-3′; MSI2a, 5′-TTCATGCTTGGCATGGGGAT-3′ and 5′-CGGGTTGGAGCCTGATCCT-3′; MSI2b, 5′-TGACATCGGTGCTCACTTCT-3′ and 5′-GTCTGCGAACGTGACGAAAC-3′; MSI2c, 5′- AGGAGCGCCAGGGTTAAAAT-3′ and 5′-CGAACGTGACGAAACCGAAG-3′; MSI2a, −d, 5′-TATGGAGGCAAATGGGAGCC-3′ and 5′-GCTATCTGGTGAGGTCTGCC-3′; TP53INP1, 5′-GCACCCTTCAGTCTTTTCCTGTT-3′ and 5′-GGAGAAAGCAGGAATCACTTGTATC-3′; and β-actin, 5′-AGTCATTCCAAATATGAGATGCGTT-3′ and 5′-TGCTATCACCTCCCCTGTGT-3′. The data were calculated using the 2^-ΔΔCT^ relative quantification method.

### Tissue microarray and immunohistochemistry

The breast cancer tissue microarray blocks were constructed using 388 blocks of primary invasive ductal carcinoma samples. Immunohistochemical (IHC) analysis of MSI2a, TP53INP1, and Ki67 expression was performed according to a previous study [18]. The anti-MSI2a (ab76148; 1:100), anti-TP53INP1 (ab202026; 1:100), and anti-Ki-67 (ab92742; 1:500) antibodies were obtained from Abcam (Cambridge, UK) and were used according to the manufacturer’s suggested dilutions and procedures. The immunostained tissue microarrays were scored semiquantitatively for MSI2a and TP53INP1 expression by two experienced pathologists with the H scoring system (ranging between 0 and 300), which is based on the staining intensity and proportion of positively stained cells. In this study, we first estimated and then used the cut-off value calculated with a receiver operating characteristic (ROC) curve to divide patients into groups of high vs. low expression of MSI2a and TP53INP1.

### Transcriptome microarray

The transcriptome profiles of differentially expressed mRNAs were performed using 30 samples (25 TNBC tissues and 5 paired ANTs) with a HiSeq 3000 platform (Illumina, San Diego, CA, USA) as described previously [18]. Briefly, RNA was isolated using TRIzol reagent, and the integrity of the RNA samples was verified using an Agilent 2100 Bioanalyzer (Agilent Technologies, Santa Clara, CA, USA). Samples with an RNA integrity number ≥ 7 were constructed into cDNA libraries and sequenced using a HiSeq 3000 platform. The differentially expressed mRNAs were defined as a greater than 1.5-fold change between the TNBC tissues and ANTs, with a *p* value < 0.05. Gene Ontology and Kyoto Encyclopedia of Genes and Genomes pathway enrichment analyses were performed using R software, version 3.2.1 (http://www.r-project.org/), to explore these differentially expressed mRNA-regulated cell processes and gene pathways.

### Cell lines and culture

Human breast cancer cell lines (MCF7, T47D, SK-BR-3, MDA-MB-231, BT20, MDA-MB-468, and Hs-578T) were kindly provided by Professor Daqiang Li of Fudan University Shanghai Cancer Center (China). The cells were cultured according to standard protocols from the American Type Culture Collection (Manassas, VA, USA).

### Plasmids and lentivirus

The siRNA constructs targeting MSI2 and TP53INP1 expression were purchased from GenePharma (Shanghai, China). The sequences targeting MSI2 were siMSI2 #1, 5′-GCAAUAUUUCGAGCAGUUUTT-3′, and siMSI2 #2, 5′-GCAACGGCCUUUACAAAUGTT-3′, while the sequences targeting MSI2a were siMSI2a #1, 5′-GCTGGACCTTTGATTGCAA − 3′, and siMSI2a #2, 5′-GACCTGTCGCCGATCTCTA-3′. The sequences targeting MSI2b were siMSI2b #1, 5′-GCTCACTTCTGTTATGTTT-3′, and siMSI2b #2, 5′-GTTATGTTTTCTCCCTCTA-3′. The sequences targeting TP53INP1 were siTP53INP1 #1, 5′-CCUGCUUUCUCCAGUUUGATT-3′, and siTP53INP1#2, 5′-CCGUGGGACUGAUGAAUUATT-3′. The scrambled negative control siRNA sequence was 5′-UUCUCCGAACGUGUCACGUTT-3′. These siRNA constructs were cloned into the lentiviral vector pLKO.1 for lentivirus production. Furthermore, the plasmids and lentiviruses for MSI2a, MSI2b and TP53INP1 were all obtained from Sbo-Bio (Shanghai, China). MSI2a, MSI2b and TP53INP1 cDNA were cloned into the p3 × flag-CMV-10 vector (Sigma-Aldrich, St. Louis, MO, USA) using a PCR-based method and were confirmed by DNA sequencing.

These plasmids were then transiently transfected into breast cancer cell lines using Lipofectamine 3000 (Invitrogen) according to the manufacturer’s instructions, while lentivirus was used to infect breast cancer cells and to obtain stable MSI2a and MSI2b overexpression and knockdown subpopulations; the cell cultures were selected by treatment with puromycin (2 μg/mL; Cayman Chemical, Ann Arbor, MI, USA) for one week.

### Cell viability and cell invasion assays

The Cell Counting Kit-8 (CCK-8), invasion, and wound-healing assays were performed according to a previous study with the standard methods [18].

### Immunofluorescence

Immunofluorescence (IF) staining was performed according to a previous study with the standard methods [18].

### Luciferase reporter assay

To explore MSI2a binding to the TP53INP1 3′-untranslated regions (3′-UTRs), we identified four potential binding sites and designed three reporter constructs with the 3′**-**UTR sequences of the TP53INP1 plasmid: TP53INP1–3′**-**UTR-A (including the S1–2 binding sites), TP53INP1–3′**-**UTR-B (including the S3–4 binding sites), and TP53INP1–3′**-**UTR (including the S1–4 binding sites). The plasmid pGL3, carrying TP53INP1–3′-UTR, TP53INP1–3′-UTR-A, TP53INP1–3′-UTR-B, TP53INP1-S3M, and TP53INP1-S4M, was constructed using PCR or PCR-based mutagenesis and then confirmed with DNA sequencing. For the luciferase reporter assay, cells were grown and cotransfected with these pGL3 plasmids, MSI2a plasmids or MSI2a RRM mutation (MSI2a-Mut) plasmids, and the *Renilla* luciferase plasmid RL-TK for 48 h. After that, total cellular protein was extracted for assaying firefly/*Renilla* luciferase activities by using the Dual-Luciferase Reporter Assay System (Promega, Madison, WI, USA) according to the manufacturer’s instructions. The relative luciferase activity was then calculated as the ratio of firefly luciferase intensity/*Renilla* luciferase intensity.

### RNA stability analysis

To evaluate the stability of TP53INP1 mRNA after knockdown of MSI2a expression in Hs-578T cells or MSI2a overexpression in MDA-MB-231 cells, the cells were plated in six-well plates, grown overnight, and then treated with actinomycin D (5 μg/mL) to inhibit gene transcription. Next, total RNA was isolated from these cell lines at the indicated time points, and the level of TP53INP1 mRNA was analyzed using qRT-PCR. The results are summarized as the percentage of the control.

### Western blotting

Western blotting was performed on extracted protein samples, according to a previous study [18], using anti-MSI2a, anti-flag, anti-TP53INP1, and anti-vinculin antibodies (all from Abcam) as well as anti-ZO-1, anti-E-cadherin, anti-N-cadherin, anti-β-catenin, anti-vimentin, anti-slug, anti-p-ERK1/2, and anti-ERK1/2 antibodies (all from Cell Signaling Technology). Vinculin was used as the loading control.

### Nude mouse tumor cell xenograft and metastasis assays

The animal study protocol was approved by the Institutional Animal Care and Use Committee of Shanghai Cancer Center, Fudan University. Six-week-old female BALB/c nude mice were purchased from the Shanghai Laboratory Animal Center (Shanghai, China) and were housed under specific pathogen-free conditions in a “barrier” facility. For the tumor cell xenograft assay, MSI2a-overexpressing, MSI2b-overexpressing and control MDA-MB-231 cells were subcutaneously injected into the flanks of the mice (2 × 10^6^ cells per mouse). After five weeks, the mice were sacrificed, and the tumor cell xenograft tissues were resected and fixed in 4% formalin for hematoxylin and eosin (H&E) staining and IHC analysis of MSI2a, TP53INP1, and Ki-67 expression.

For the experimental metastasis assay, MSI2a-overexpressing, MSI2b-overexpressing and control MDA-MB-231 cells (2.0 × 10^6^ cells per mouse) were injected into the tail veins of another group of mice in 200 μL of phosphate-buffered saline. After 7 weeks, the mice were sacrificed, and their lungs were removed to assess the number of tumor nodules on the lung surfaces.

### MSI2a RNA immunoprecipitation and qRT-PCR

The Magna RIP Kit (17–701; EMD Millipore, Billerica, MA, USA) was used for the RIP assay in Hs-578 t cells with high MSI2 expression, according to the manufacturer’s protocol. Briefly, Hs-578 t cells were grown in four 15-cm dishes for three days and then washed three times with ice-cold phosphate-buffered saline and centrifuged to collect the cells. The cells were collected and resuspended in 200 μL of RIP buffer and then incubated with magnetic protein A/G beads conjugated to the indicated antibodies (anti-MSI2, ab114083, specific for MSI2a) at 4 °C for 3 h. A 100-μL aliquot of the supernatant was diluted with 900 μL of RIP buffer and then treated with proteinase K solution to isolate MSI2a protein-associated RNA from the eluted immunocomplexes. RNA was then isolated and extracted by using the phenol/chloroform method for RIP-Seq, and the level of TP53INP1 mRNA in the RIP complex was assayed by using qRT-PCR.

### mRNA sequencing and data analysis

Stable MSI2a-overexpressing and MSI2a-knockdown TNBC cells were subjected to RNA-Seq to profile differentially expressed mRNAs. Briefly, total RNA was isolated from these cells using TRIzol (Invitrogen) and then subjected to RNA-Seq. We utilized a fold change > 1.5 with *p* < 0.05 as the threshold to identify differentially expressed mRNAs in these cells vs. control cells.

### Statistical analysis

All in vitro experiments were performed in triplicate and repeated independently at least three times. The data were expressed as the mean ± standard deviation, and the differences among the groups were analyzed using Student’s *t* test or one-way analysis of variance. The association between two gene expression levels was determined using Pearson correlation analysis. Kaplan-Meier curves and the log-rank test were performed to analyze patient survival stratified by gene expression. A value of *p <* 0.05 was considered statistically significant. Statistical analyses were performed using SPSS 20.0 (SPSS, Chicago, IL, USA) or GraphPad Prism 7.0 (GraphPad Software, La Jolla, CA, USA).

## Results

### MSI2 isoforms downregulation in TNBC and its association with poor prognosis

**Fig. 1:**
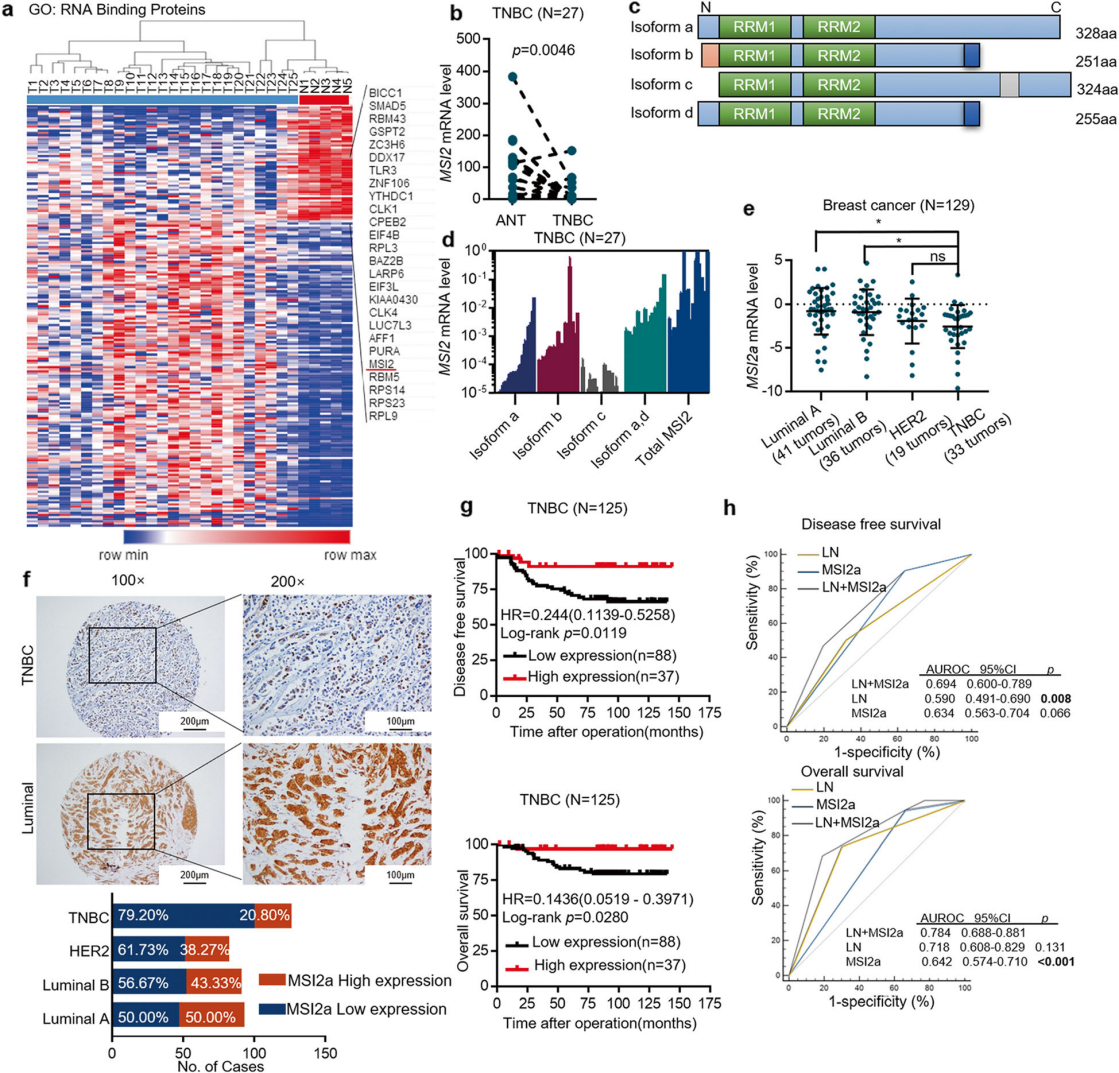
MSI2 downregulation in TNBC tissues and its association with poor TNBC prognosis. **a** Heatmap of differentially expressed mRNAs related to nucleotide binding between TNBC tissues and ANTs (> 1.5-fold and *p* < 0.05). **b** qRT-PCR. MSI2 mRNA expression levels in 27 pairs of TNBC and normal tissues. **c** Structure of the MSI2 isoforms. Isoform a is the canonical MSI2 isoform. The N- and C-termini of isoform b are distinct from those of isoform a. The N-terminus of isoform c lacks 4 aa compared with the N-terminus of isoform a. Isoform d contains the same N-terminus as isoform a and the same C-terminus as isoform b. **d** Expression analysis by qRT-PCR of MSI2 isoforms a-d mRNA in 27 TNBC tumor tissues. **e** qRT-PCR. MSI2a mRNA expression levels across different breast cancer types. **f** Immunohistochemistry. Representative tissue microarray cores of TNBC or luminal breast cancer samples after immunostaining with an anti-MSI2a antibody. Images were magnified with a 10× or 20× objective. Scale bar, 100 μm and 200 μm, respectively. **g** Kaplan-Meier survival curves comparing OS and DFS in patients stratified by low vs. high MSI2a mRNA levels in breast cancer tissues. **h** ROC curves of DFS and OS showing the area under the ROC curve (AUROC) of the combined MSI2a expression and lymph node (LN) status versus AUROCs of MSI2 expression alone or LN status alone

In this study, transcriptome microarray analysis of differentially expressed mRNAs between 25 TNBC tissues and 5 ANTs (Table S1–S2) was performed. In total, 145 upregulated and 55 downregulated RBP genes were identified in TNBC vs. ANTs (Fig. 1a). Among them, MSI2 was downregulated in TNBC, which is consistent with results based on the GSE58135 dataset (Fig. S1a); therefore, we selected MSI2 for further investigation. First, we measured the total MSI2 mRNA level in 27 paired frozen TNBC tissues and ANTs and found it to be significantly downregulated in 88.89% (24/27) of TNBC tissues vs. ANTs (*p* = 0.0046; Fig. 1b, and Table S1). TCGA database suggested that MSI2 expression in TNBC was reduced compared to that in other subtypes (Fig. S1b). The spectrum of MSI2 isoforms (Fig. 1c) was then determined by PCR in 27 TNBC tissues (Fig. 1d). Because there were no specific primers for MSI2 isoform d (MSI2d), we used primers capable of detecting isoforms a (MSI2a) and d. The results showed that there was higher expression of isoform b (MSI2b, mean = 0.01011) than MSI2a (mean = 0.001323) and that isoform c (MSI2c, mean = 1.58e-005) exhibited the lowest expression, though there was no significant difference among these values. The expression of total MSI2a and MSI2d (mean = 0.009765) was relatively high, which may indicate that MSI2d also has a certain expression in TNBC. Thus, we mainly focused on the functions of MSI2a and MSI2b in TNBC in this study. Transcript analysis of MSI2 isoforms in our RNA sequencing (RNA-Seq) data also indicated that MSI2a was significantly downregulated in TNBC tissues vs. ANTs (Fig. S1c). Next, we compared the mRNA levels of MSI2a and MSI2b between 27 paired frozen TNBC tissues and ANTs and found that the expression of both in TNBC tissues was significantly decreased compared to that in ANTs (Fig. S1d). We further assessed the expression of MSI2a and MSI2b by qRT-PCR in another group of 129 frozen breast cancer tissues and found that the expression of the former in TNBC tissues was downregulated compared with that in luminal A and luminal B breast cancer tissues (Fig. 1e), which is consistent with TCGA database. Next, with ROC analysis, we divided our patients into two groups: tumors with low MSI2a expression (65; 50.38%) and tumors with high MSI2a expression (64; 49.62%). Downregulation of MSI2a mRNA expression was associated with a higher histological grade of breast cancer (*p* = 0.042, Table S3) and poor overall survival (OS, *p* = 0.0073; Fig. S1e and Table S4). When analyzing MSI2b expression, we observed that MSI2b in TNBC tissues was downregulated compared with that in luminal A and luminal B breast cancer tissues (Fig. S1f), though ROC analysis indicated that MSI2b expression was not sufficient to divide our patients into two groups to predict patient survival (Fig. S1g).

Currently, commercialized antibodies for MSI2 are directed against isoform a. Therefore, we performed immunohistochemistry to assess MSI2a protein expression in 388 breast cancer patients, including 125 TNBC patients. Cytoplasmic MSI2a staining was observed in 375 (96.65%) samples. 388 cases were divided into low- (246; 63.40%) and high-expression groups (142; 36.60%) according to the MSI2a protein expression level based on the ROC curve. We found that tumors with low MSI2a expression had a higher histological grade (*p* = 0.038) and were more likely to be TNBC (*p <* 0.001; Fig. 1f and Table S5.). Moreover, tumors with low MSI2a expression had significantly high rates of cancer recurrence (*p <* 0.001) and distant metastasis (*p* = 0.001; Table S5.). However, there was no difference observed between high vs. low MSI2a expression in terms of patient age, number of positive lymph nodes, or tumor size. Patients with high MSI2a expression exhibited significantly better disease-free survival (DFS) (*p <* 0.001) and OS (*p <* 0.001) than patients with low MSI2a expression (Fig. S1h). Multivariate analysis indicated that MSI2a expression was an independent predictor of DFS and OS of breast cancer patients (Table S6).

In addition, we assessed the prognostic significance of MSI2a expression in 125 TNBC patients and found that TNBC tissues with low MSI2a expression were more likely to have a high histological grade (*p* = 0.004) and more distant metastases (*p* = 0.004; Table S7), whereas patients with high MSI2a expression exhibited significantly longer DFS (*p* = 0.012, hazard ratio (HR) = 0.244, 95% CI, 0.11–0.52) and OS (*p* = 0.028; HR = 0.14, 95% CI, 0.052–0.39; Fig. 1g and Table S8). Multivariate analysis indicated that MSI2a expression was an independent predictor for DFS of TNBC patients (Table S8); moreover, the combination of the MSI2 expression level with the lymph node status could better predict prognosis than each factor individually (Fig. 1h). Collectively, our data demonstrated that MSI2a expression was frequently downregulated in TNBC and associated with poor DFS and OS in breast cancer and TNBC patients.

### MSI2a and MSI2b differentially regulate TNBC cell proliferation and metastasis in vitro and in vivo

**Fig. 2:**
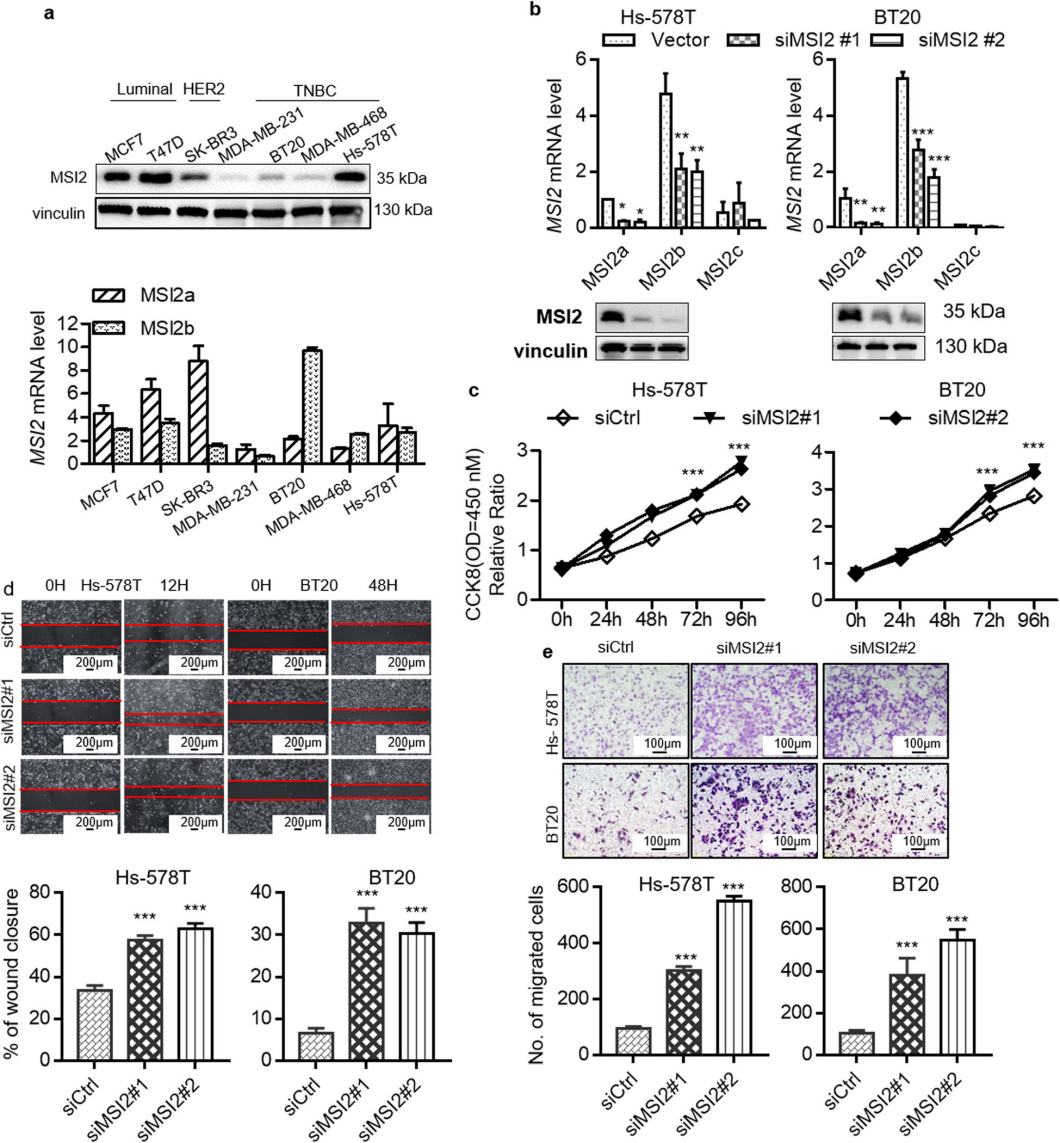
MSI2 knockdown promoted TNBC cell growth and migration in vitro*.***a** Western blot and qRT-PCR assays. MSI2 expression was detected in multiple breast cancer cell lines. **b** Western blot and qRT-PCR assays. The efficiency of MSI2 knockdown in Hs-578T and BT20 cells. **c** CCK-8 assay for cell viability. Cell proliferation was assessed after MSI2 knockdown in Hs-578T and BT20 cells. **d** Wound-healing assay. Images of the wound-healing assay data showing the wound-healing ability in MSI2-knockdown Hs-578T and BT20 cells. Scale bar, 200 μm. **e** Transwell migration assay. Images showing the migration capacity in MSI2-knockdown Hs-578T and BT20 cells. Scale bar, 100 μm.

**Fig. 3:**
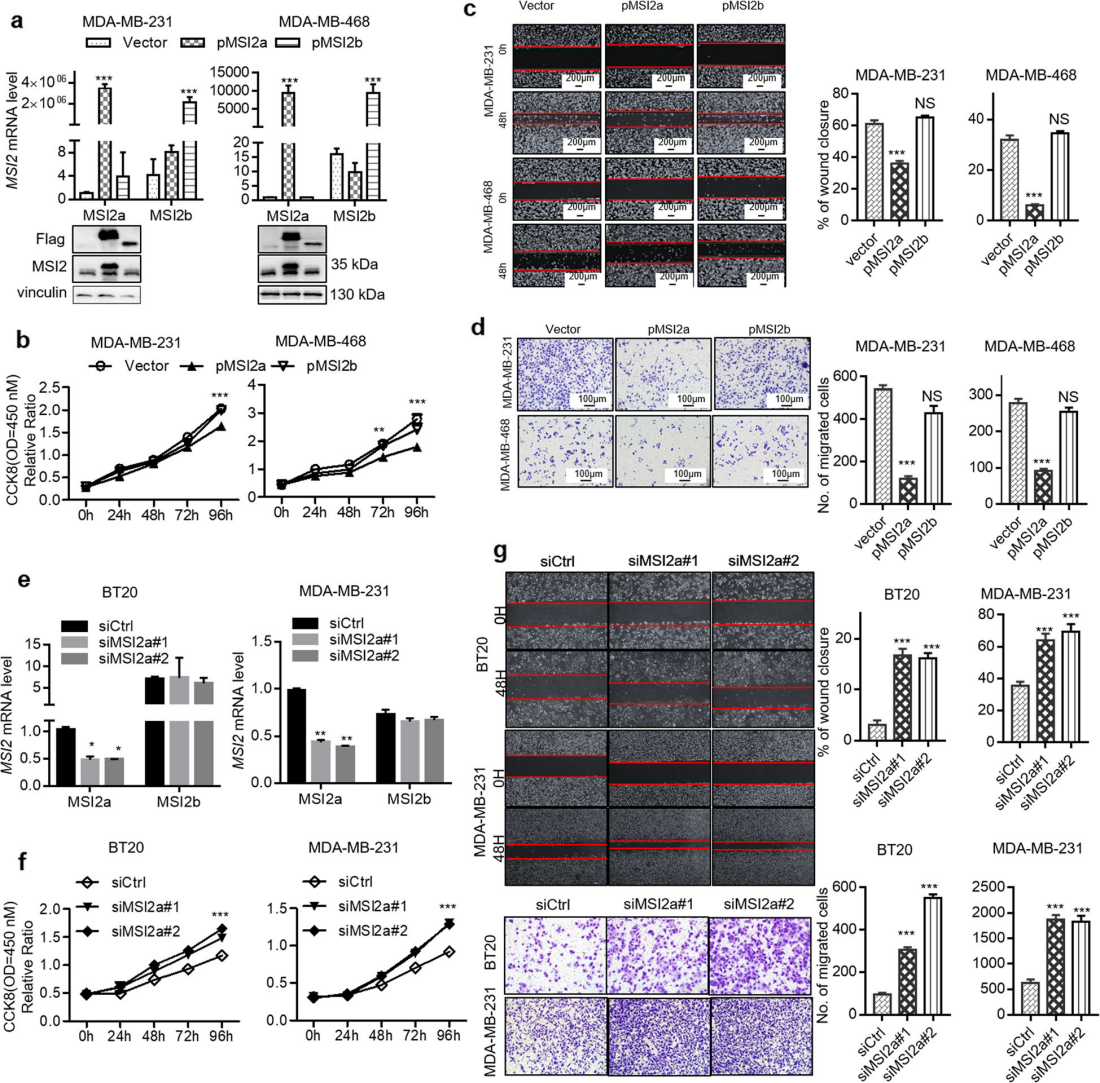
MSI2a negatively regulates TNBC cell growth and migration in vitro*.***a** Western blot and qRT-PCR assays. In MDA-MB-231 and MDA-MB-468 cells, MSI2a transfection significantly increased MSI2a mRNA and protein expression but did not affect MSI2b expression. MSI2b transfection significantly increased MSI2b mRNA and protein expression but did not affect MSI2a expression. **b** CCK-8 assay for cell viability. Cell proliferation was assessed after MSI2a and -b overexpression in MDA-MB-231 and MDA-MB-468 cells **c** Wound-healing assay. Images of the wound-healing assay data showing the wound-healing ability in MSI2a- and -b-overexpressing MDA-MB-231 and MDA-MB-468 cells. Scale bar, 200 μm. **d** Transwell migration assay. Images showing the migration capacity in MSI2a- and -b-overexpressing MDA-MB-231 and MDA-MB-468 cells. Scale bar, 100 μm. **e** qRT-PCR assays. The efficiency of MSI2a knockdown in BT20 and MDA-MB-231 cells. **f** CCK-8 assay for cell viability. Cell proliferation was assessed after MSI2a knockdown in BT20 and MDA-MB-231 cells. **g **Transwell migration assay and wound-healing assay. Images showing the migration capacity and wound-healing ability of MSI2a-knockdown BT20 and MDA-MB-231 cells

**Fig. 4:**
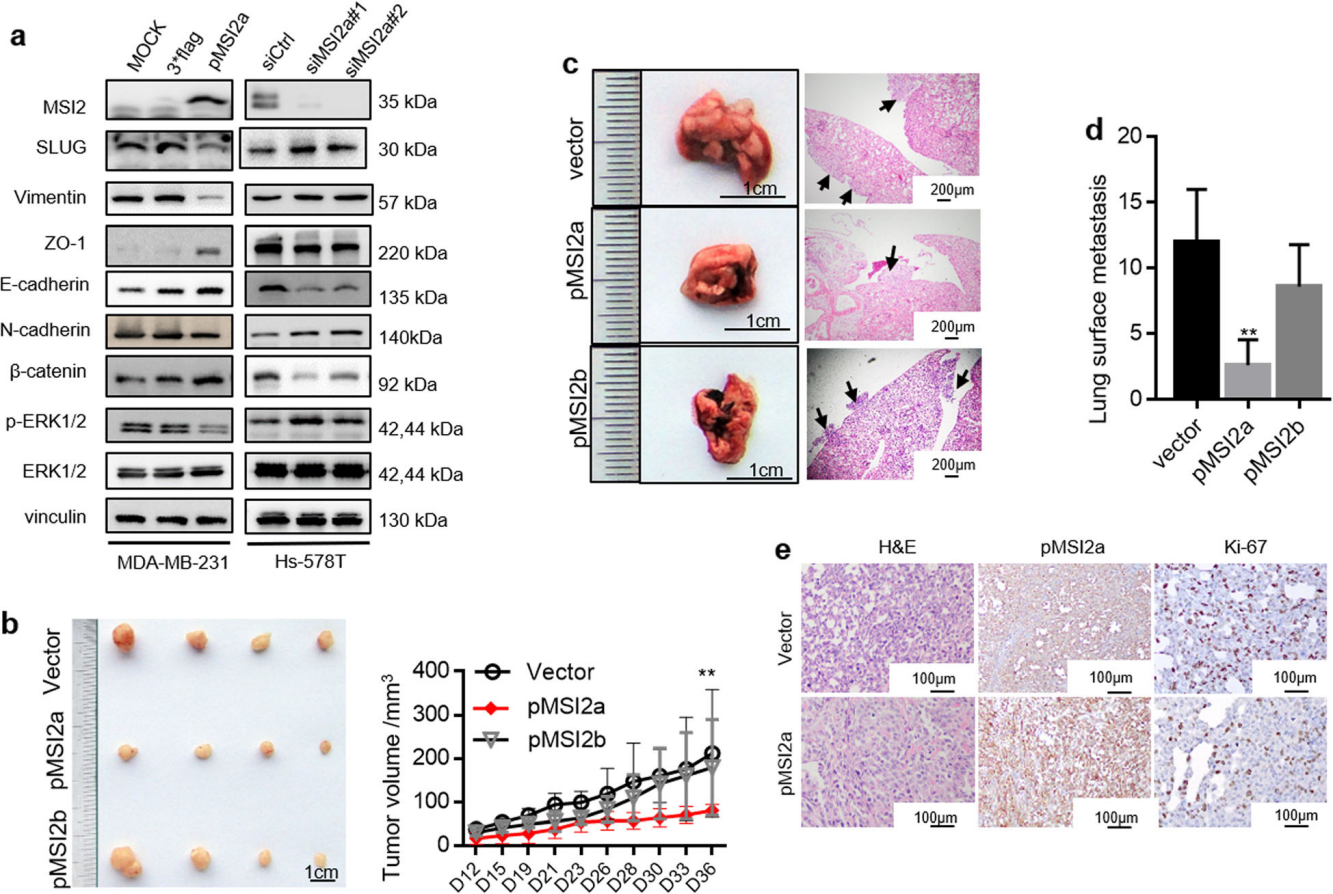
MSI2a negatively regulates TNBC cell growth and migration in vivo. **a** Western blot assays of SLUG, ZO-1, vimentin, E-cadherin, N-cadherin, β-catenin, p-ERK1/2, and ERK1/2 expression in MDA-MB-231 and Hs-578T cells. **b** Tumorigenicity assay of MSI2a-overexpressing and -b-overexpressing MDA-MB-231 cells (2 × 10^6^) after subcutaneous injection in the flanks of nude mice (*n* = 4). The mice and tumors are shown. The quantification of tumor volume is shown on the right side. Scale bar, 1 cm. **c** Representative images of nude mouse lungs detecting MDA-MB-231 cells at 50 days after tumor cells were injected into the tail vein (scale bar, 1 cm) and H&E staining of the lungs (right; scale bar, 100 mm); arrows, metastatic foci. **d** Statistical analysis of the numbers of lung surface metastases using Student’s *t* test. *n* = 5. **e** H&E and MSI2 and Ki67 staining of xenograft tissues of mice injected with MSI2a-overexpressing MDA-MB-231 cells. Scale bar, 100 μm. *p < 0.05, **p < 0.01, ***p < 0.001

To study the roles of MSI2 isoforms in the regulation of TNBC cell proliferation and metastasis, we first detected the mRNA expression levels of MSI2a and MSI2b and the protein expression level of MSI2a in seven different breast cancer cell lines (Fig. 2a). We then knocked down total MSI2 expression using siRNAs targeting all MSI2 isoforms in Hs-578T and BT20 cells, which have high MSI2 expression (Fig. 2b). We tested cell viability by CCK-8 assay and found that knockdown of total MSI2 expression enhanced the viability of Hs-578T and BT20 cells (Fig. 2c). Furthermore, knockdown of total MSI2 expression promoted wound healing and migration in these cells (Fig. 2d and e). To further investigate which isoform is responsible for the function of MSI2, MDA-MB-231 and MDA-MB-468 cells were transduced with lentiviral vectors expressing MSI2a and MSI2b (Fig. 3a). MSI2a overexpression reduced cell viability (Fig. 3b) and suppressed tumor cell wound healing (Fig. 3c) and migration (Fig. 3d) in MDA-MB-231 and MDA-MB-468 cells. Consistent with this, MSI2a knockdown (Fig. 3e) using isoform-specific siRNAs enhanced the viability (Fig. 3f) and promoted migration and wound healing (Fig. 3g) of BT20 and MDA-MB-231 cells. Conversely, MSI2b expression showed no significant effect on the TNBC phenotype, nor did MSI2b knockdown (Fig. S2a-c). We next investigated EMT-related gene expression and found that MSI2a overexpression significantly downregulated the expression of the mesenchymal markers SLUG, N-cadherin and vimentin, and upregulated that of the epithelial markers E-cadherin, ZO-1, β-catenin, and phosphorylated extracellular signal-regulated kinase 1/2 (p-ERK1/2) (Fig. 4a and Fig. S2d).

To further validate our observations in vivo, we injected MSI2a- and MSI2b-transfected MDA-MB-231 cells into either the subcutis or the caudal vein of nude mice in groups of four. Consistent with the effect of altered MSI2a and MSI2b expression on MDA-MB-231 cells in vitro, compared with the control group, the MSI2a overexpression group had a significant tumor growth inhibition (Fig. 4b and Fig. S2e) and abrogated lung metastases (Fig. 4c). MSI2b overexpression had no significant effect (Fig. 4b and c). At the protein level, MSI2a overexpression reduced Ki-67 expression in MDA-MB-231 xenografts (Fig. 4e).

Taken together, these data indicate that MSI2a expression inhibited the proliferation and invasiveness of TNBC by suppressing tumor cell EMT, while MSI2b showed no significant function in TNBC progression.

### MSI2a interacts with TP53INP1 mRNA and inhibits ERK activation

**Fig. 5:**
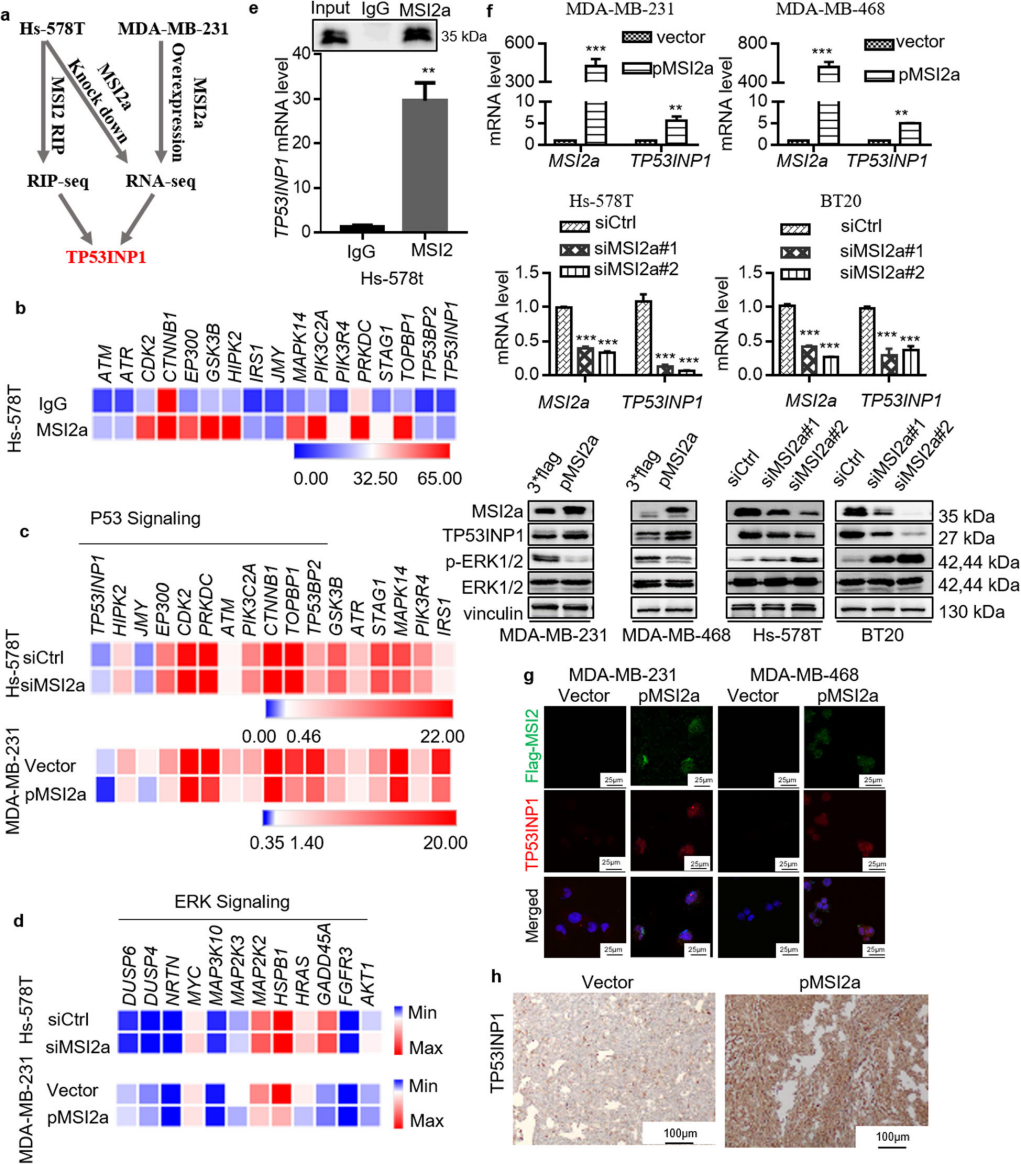
MSI2a binds to TP53INP1 mRNA, increasing TP53INP1 expression and suppressing ERK1/2 activation. **a** Diagrams of the MSI2-targeted mRNA TP53INP1. **b** Heatmap showing that 17 mRNAs were associated with p53 signaling and enriched by MSI2a. **c** Heatmap showing the expression levels of 17 mRNAs associated with p53 signaling after RNA-Seq analysis. **d** Heatmap showing the changes in gene expression associated with ERK signaling after RNA-Seq analysis. **e** RIP assay was performed using cell extracts prepared from Hs-578T cells with an MSI2a-specific antibody or isotype-matched IgG. The level of TP53INP1 mRNA was quantified by using qRT-PCR, while Western blotting showed the specificity of the MSI2a antibody. **f** qRT-PCR analysis of TP53INP1 mRNA and Western blot analysis of the MSI2, TP53INP1, p-ERK1/2, and ERK1/2 proteins after MSI2a overexpression or knockdown in these TNBC cells. **g** MSI2a and TP53INP1 expression levels were analyzed in MSI2a-overexpressing MDA-MB-231 and MDA-MB-468 cells by immunofluorescence staining. Green and red represent MSI2a and TP53INP1, respectively. **h** TP53INP1 staining of xenograft tissues of mice injected with MSI2a-overexpressing MDA-MB-231 cells. Scale bar, 100 μm. *p < 0.05, **p < 0.01, ***p < 0.001

To elucidate the potential mechanism of MSI2a in TNBC, we performed RNA immunoprecipitation and sequencing (RIP-Seq) in Hs-578T cells to profile MSI2a-binding transcripts and RNA-Seq using MSI2a-overexpressing MDA-MB-231 cells and MSI2a-knockdown Hs-578T cells to profile potential target genes of MSI2a (Fig. 5a). Our RIP-Seq data identified 1547 target genes of MSI2a with a greater than two-fold change (*p <* 0.05; Table S9). Ingenuity pathway analysis of these enriched genes revealed that the TP53 signaling pathway was among the top three most significant signaling pathways involved in mediating MSI2a functions in TNBC cells (Fig. 5b and Fig. S3a). Moreover, the RNA-Seq data showed that all 17 genes related to p53 signaling were upregulated in MSI2a-overexpressing MDA-MB-231 cells, although only TP53INP1, HIPK2, JMY, and EP300 were downregulated in MSI2a-silenced Hs-578T cells (Fig. 5c and Table S10). Our RNA-Seq data also revealed that MSI2a significantly downregulated the expression of genes related to ERK signaling (Fig. 5d). We then performed RIP-qRT-PCR or Western blot assays to validate MSI2a-mediated regulation of TP53INP1 expression and their interaction (Fig. 5e). We found that MSI2a overexpression increased the mRNA and protein levels of TP53INP1 but decreased p-ERK1/2 expression in MDA-MB-231 and MDA-MB-468 cells, whereas knockdown of MSI2a expression had the opposite effects in Hs-578T and BT20 cells (Fig. 5f). Furthermore, IF staining confirmed that MSI2a overexpression enhanced TP53INP1 expression in MDA-MB-231 and MDA-MB-468 cells (Fig. 5g). In addition, high MSI2a expression in tumor cell xenografts was associated with strong TP53INP1 staining (Fig. 5h). An association between the MSI2a and TP53INP1 mRNA levels was also observed in our cohorts of breast cancer tissues (Fig. S3b), the CCLE breast cancer cell lines (Fig. S3c), TCGA breast cancer dataset (Fig. S3d), and the GESA76250 TNBC samples (Fig. S3e). These findings suggest that MSI2a interacts with TP53INP1 to upregulate TP53INP1 expression but downregulate ERK activity.

### MSI2a directly binds to the TP53INP1 mRNA 3′-UTRs and promotes TP53INP1 mRNA stability in TNBC cells

**Fig. 6:**
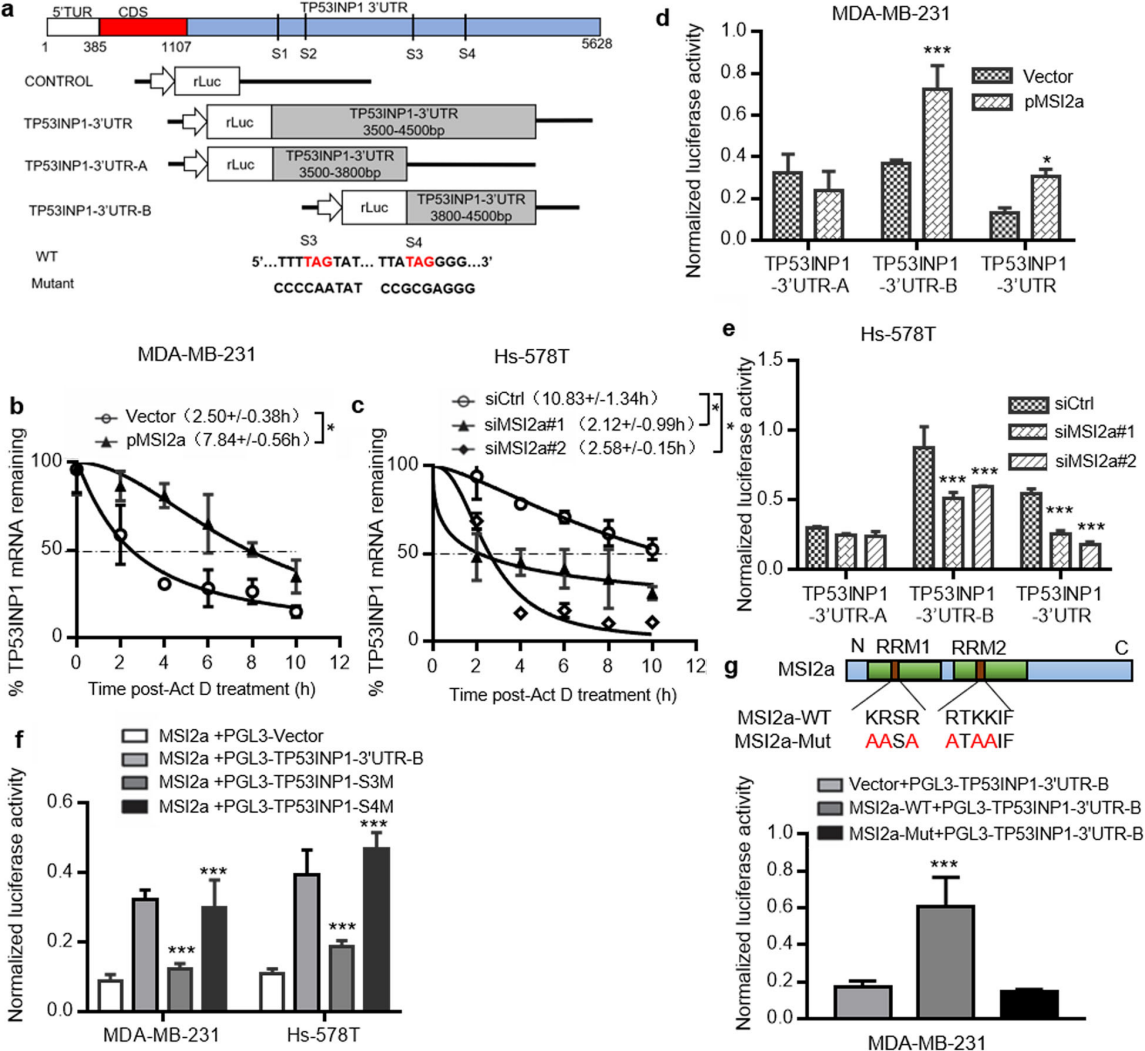
MSI2a binds to TP53INP1 mRNA and increases TP53INP1 mRNA stability and protein expression. **a** Schematic map of construction of the 3′**-**UTR reporter constructs for TP53INP1 that were used for a dual-luciferase assay. TP53INP1 mRNA stability curves plotted using qPCR expression versus time. **b-d** Luciferase reporter assay. **(b)** MDA-MB-231 cells transfected with MSI2a plasmids and **(c)** Hs-578 T cells transfected with MSI2a siRNA were treated with actinomycin D (5 mg/mL) for the indicated periods of time. (**d)** MDA-MB-231 cells were cotransfected with MSI2-overexpressing plasmids, *Renilla* luciferase 3′**-**UTR constructs, and a control firefly luciferase control vector. **e** Hs-578 T cells were cotransfected with MSI2a-specific siRNAs, *Renilla* luciferase 3′**-**UTR constructs, and a control firefly luciferase control vector. **f** Luciferase reporter assay. MDA-MB-231 and Hs-578 T cells were cotransfected with MSI2a-overexpressing plasmids, *Renilla* luciferase, 3′**-**UTR constructs, and a control firefly luciferase control vector. *Renilla* luciferase activity following MSI2a overexpression was determined. **g** Luciferase reporter assay. Schematic map of construction of the MSI2a-Mut plasmids. MDA-MB-231 cells were cotransfected with MSI2a or MSI2a-Mut plasmids, *Renilla* luciferase, 3′**-**UTR constructs, and a control firefly luciferase control vector. *Renilla* luciferase activity was determined. The data were normalized to firefly luciferase activity. *p < 0.05, **p < 0.01, ***p < 0.001

It has been previously shown that MSI2 primarily recognizes mRNA 3′-UTRs enriched with multiple UAG motifs to regulate protein translation, mRNA stabilization, and alternative splicing [14]. We identified four possible MSI2a-binding sites in the TP53INP1 3′**-**UTR (Fig. 6a). To confirm their interactions, we first treated tumor cells with actinomycin D to assess the rate of TP53INP1 mRNA decay and found that the half-life of TP53INP1 mRNA was significantly extended in MSI2a-overexpressing TNBC cells (Fig. 6b) but significantly shortened in MSI2a-knockdown TNBC cells (Fig. 6c). Luciferase reporter assays revealed that MSI2 overexpression significantly enhanced the luciferase activity of the TP53INP1–3′**-**UTR-B and TP53INP1–3′**-**UTR constructs (Fig. 6d), whereas MSI2a knockdown significantly decreased their activity (Fig. 6e). However, when using a mutated S3- or S4-binding site in region B (PGL3-TP53INP1–3′**-**UTR-S3M and PGL3-TP53INP1–3′**-**UTR-S4M, respectively), the mutated S3-binding site was shown to have significantly reduced luciferase activity in MSI2a-overexpressing Hs-578 T and MDA-MB-231 cells (Fig. 6f). When using a RRM-mutated MSI2a plasmid, luciferase reporter assays showed that compared with MSI2a overexpression in MDA-MB-231 cells, MSI2a-Mut overexpression significantly reduced the luciferase activity of TP53INP1–3′-UTR-B (Fig. 6g). These data suggest that the RRMs of MSI2a can directly interact with TP53INP1 mRNA and regulate transcript stability.

### MSI2a inhibition of TNBC cell metastasis in a TP53INP1/ERK-mediated manner

**Fig. 7:**
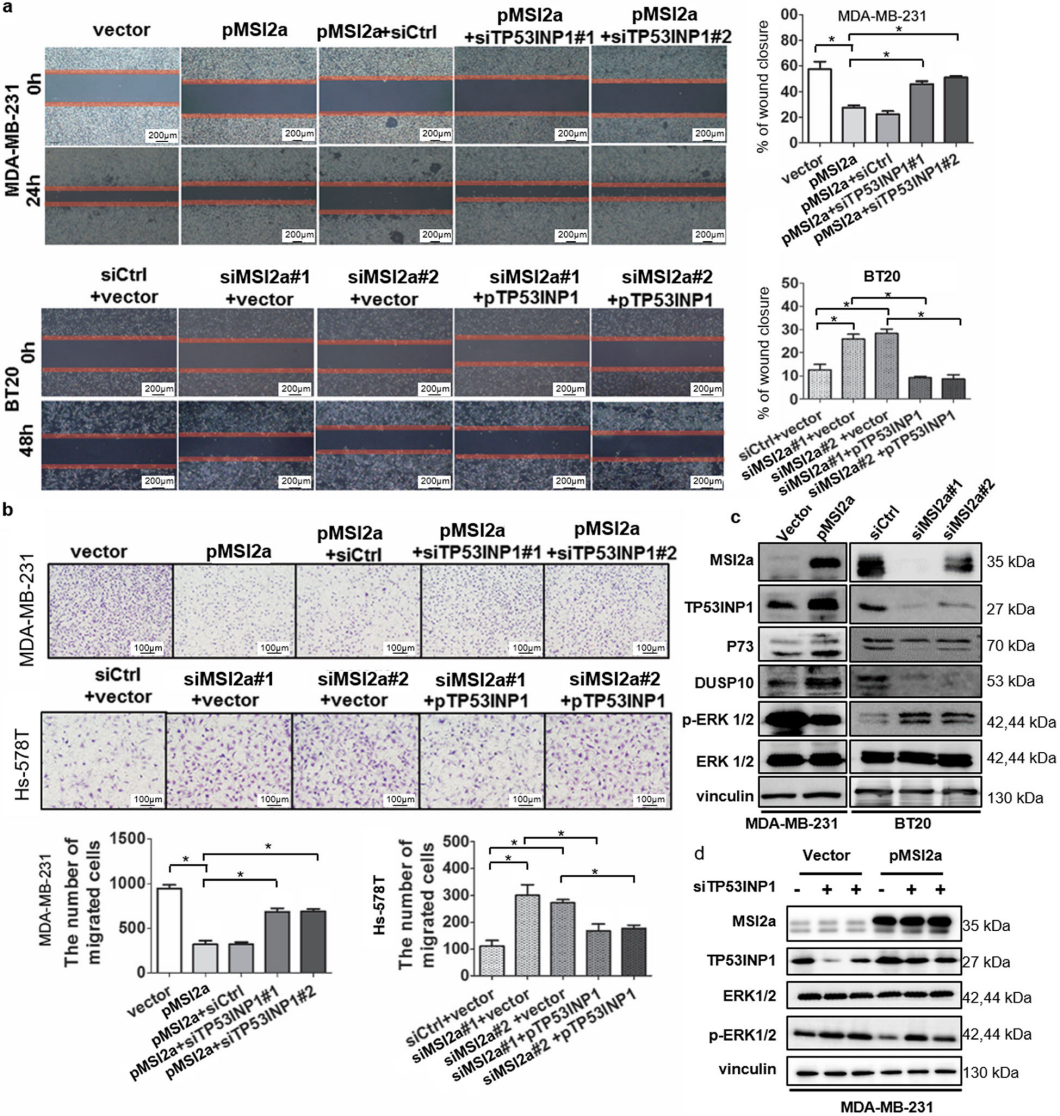
MSI2a inhibited TNBC progression in a TP53INP1-mediated manner. **a** Wound-healing assays. Representative images and quantification of the assay results show the wound-healing capacity of MSI2a-overexpressing MDA-MB-231 cells transfected with siTP53INP1 or MSI2a-knockdown Hs-578T cells transfected with TP53INP1 plasmids, respectively. Scale bar, 200 μm. **b** Representative images and quantification of the number of migrated cells of MDA-MB-231 cells coexpressing empty vector or MSI2a and control siRNA or siTP53INP1 as well as Hs-578T cells coexpressing control siRNA or siMSI2a and empty vector or TP53INP1. Scale bar, 100 μm. **c** MSI2a-overexpressing MDA-MB-231 cells and MSI2a-knockdown BT20 cells were analyzed by Western blot with anti-MSI2, anti-TP53INP1, anti-P73, anti-DUSP10, anti-ERK, and anti-p-ERK antibodies. Vinculin was used as a control. **d** MDA-MB-231 cells coexpressing empty vector or MSI2a and control siRNA or siTP53INP1 were analyzed by Western blot with anti-MSI2a, anti-TP53INP1, anti-ERK, and anti-p-ERK antibodies. Vinculin was used as a control. *p < 0.05

To confirm the role of TP53INP1 in mediating the anti-TNBC activity of MSI2, we first assessed the effects of TP53INP1 on TNBC cells. We found that ectopic TP53INP1 expression inhibited MDA-MB-231 cell migration and wound healing but that knockdown of TP53INP1 expression enhanced the invasiveness of Hs-578T  cells (Fig. S4a and b). We then performed a rescue experiment and found that knockdown of TP53INP1 expression reversed the inhibitory effect of MSI2a on MDA-MB-231 cell metastasis and that MSI2a knockdown-induced cell migration and wound healing were partially restored by TP53INP1 overexpression in BT20 and Hs-578T cells (Fig. 7a and b). Downregulation of TP53INP1 has been reported to promote hepatocellular carcinoma cell metastasis through the p73/DUSP10 pathway and activation of ERK1/2 [19]. As our RIP-Seq data showed that MSI2a affected ERK signaling pathway activation, we assessed whether TP53INP1 had similar functions and found that TP53INP1 negatively regulated ERK activation by altering the levels of P73 and DUSP10 in TNBC (Fig. S4c). Moreover, ectopic MSI2a expression increased the TP53INP1, P73, and DUSP10 levels but decreased the p-ERK levels, whereas knockdown of MSI2a had the opposite effects (Fig. 7c). In addition, MSI2a expression-inhibited ERK activation was rescued by TP53INP1 knockdown (Fig. 7d).

Furthermore, treatment of MSI2a-transfected TNBC cells with the ERK1/2 inhibitor U0126 reduced the scratch closure rate (Fig. S5a) and cell migration (Fig. S5b) in MSI2a-knockdown Hs-578T and BT20 cells. These data suggest that MSI2 inhibits TNBC metastasis through increased TP53INP1 expression and suppression of ERK1/2 activation.

### MSI2a/TP53INP1 as a biomarker panel in breast cancer

**Fig. 8:**
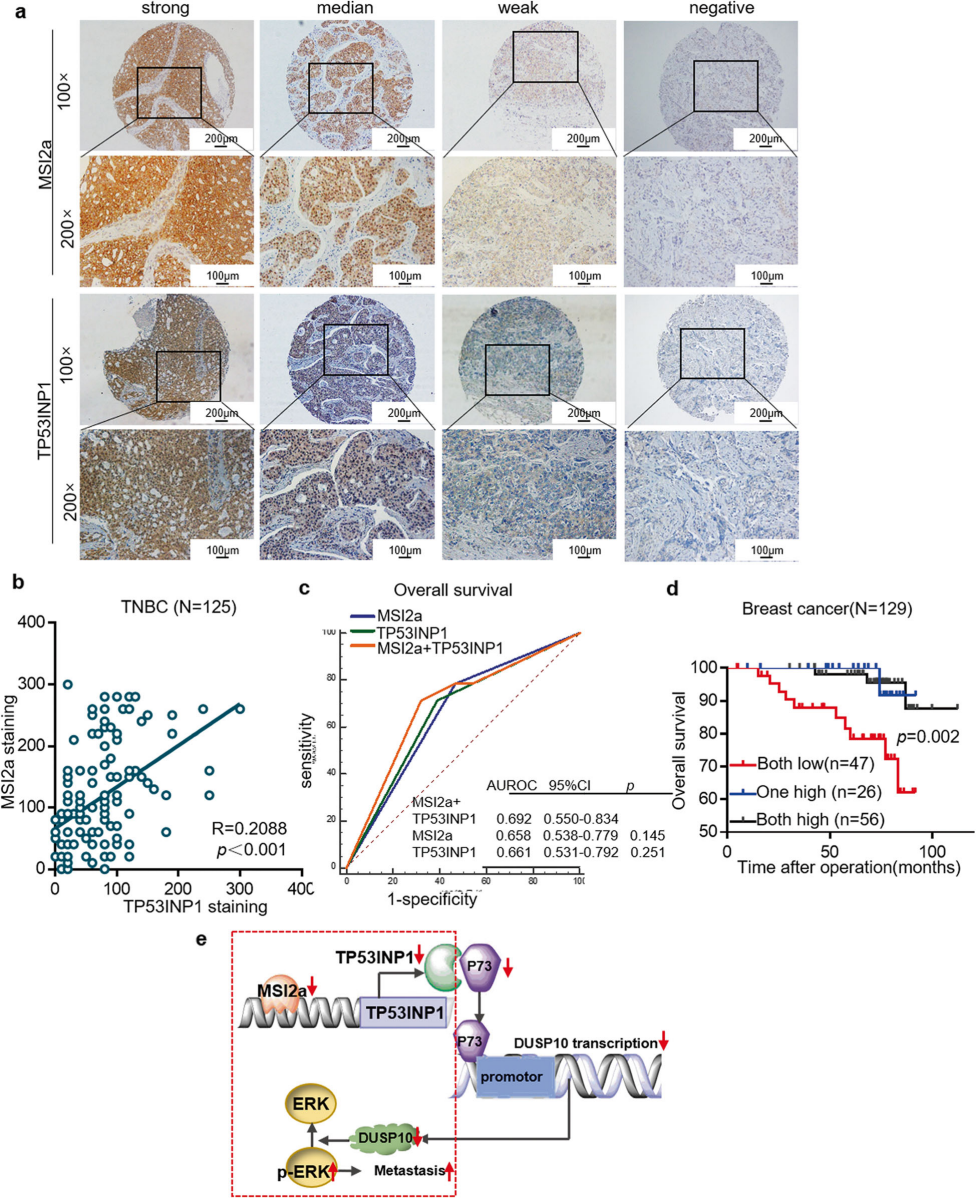
Association of TP53INP1 expression with MSI2 expression. **a** Immunohistochemistry. TNBC tissues were subjected to immunohistochemical staining of MSI2 and TP53INP1. Representative images were acquired with 10× and 20× objectives. Scale bar, 100 μm and 200 μm. **b** Plot depicting the positive correlation between MSI2 protein expression and TP53INP1 protein expression in TNBC. **c** Kaplan-Meier survival curves comparing OS of breast cancer combined with MSI2 mRNA and TP53INP1 mRNA expression. **d** Kaplan-Meier survival curves comparing OS of breast cancer combined with MSI2 mRNA and TP53INP1 mRNA expression. **e** A schematic model of MSI2/TP53INP1 function during TNBC metastasis. Normally, MSI2a interacts with the 3’UTR of TP53INP1 mRNA, leading to the stabilization of TP53INP1 mRNA, which in turn ensures complete p-ERK inactivation. In the case of MSI2a dysregulation in TNBC, TP53INP1 mRNA becomes unstable, resulting in decreased TP53INP1, inevitably causing increased p-ERK and TNBC metastasis

TP53INP1 protein expression in 125 TNBC samples was positively associated with MSI2a expression (R = 0.209, *p* < 0.001, Fig. 8a-b), although TP53INP1 protein expression was not associated with DFS or OS in TNBC patients (Fig. S6a). Furthermore, breast cancer with low mRNA expression levels of both MSI2a and TP53INP1 correlated with the worst OS (*p* = 0.002; Fig. 8c–d). TP53INP1 expression was significantly downregulated in 81.48% (22/27) of TNBC tissues vs. ANTs (Fig. S6b) and other breast cancer subtypes in TCGA dataset (Fig. S6c). Low mRNA levels of TP53INP1 were associated with a high histological grade of breast cancer (Table. S3), whereas high TP53INP1 mRNA levels were significantly associated with a better OS (*p* = 0.004, HR = 0.21, 95% CI, 0.072–0.64) than that of the low TP53INP1 expression group (Fig. S6d). Overall, MSI2a and TP53INP1 may serve as a biomarker panel for predicting breast cancer outcomes in the clinic.

## Discussion

Previous studies have demonstrated that posttranscriptional regulation of RBP expression plays a crucial role in regulating TNBC initiation and progression [20–22]. Further investigations and a better understanding of RBP functions in TNBC may help in the identification of biomarkers that can predict prognosis and treatment response and in the evaluation of potentially novel targets to design therapeutic approaches for breast cancer. In our current study, we first identified 200 differentially expressed RBPs in TNBC tissues vs. ANTs and found MSI2 to be downregulated in TNBC.

MSI2 is a potent oncogene that plays key roles in hematopoietic stem cell homeostasis and malignant hematopoiesis [23]. MSI2 ablation resulted in failure to maintain hematopoietic stem cells and engraftment [24]. MSI2 alteration also promoted the transformation of intestinal cells into tumor cells by acting as a pleiotropic inhibitor of various known intestinal tumor suppressors, such as PTEN [25]. Nevertheless, other studies demonstrated that loss of MSI2 stimulated epithelial cell migration, increased the number of focal adhesions and compromised cell growth [26]. These findings suggest that the role of MSI2 in cancer is complex. Furthermore, the different alternatively spliced MSI2 isoforms were also reported to have specific roles. The canonical isoform MSI2a was reported to activate target mRNA translation during the maturation of Xenopus oocytes and during the differentiation of mammalian cells in culture via phosphorylation of two conserved serine residues, while truncated MSI2b lacking the two sites failed to promote target mRNA translation and was nonpermissive for the maturation of Xenopus oocytes [17]. Additionally, MSI2a, but not isoform b, enhanced the self-renewal capacity of ESCs [16]. In breast cancer, a previous study showed that MSI2 helped to maintain the epithelial state and repressed breast cancer cell EMT and that MSI2 overexpression induced the expression of the epithelial marker E-cadherin and reduced that of the mesenchymal marker SLUG, whereas knockdown of MSI2 expression in breast cancer cells promoted loss of epithelial identity [14]. Nonetheless, MSI2 was also reported to act as an upstream regulator of ESR1, leading to luminal breast cancer proliferation and invasion, whereas MSI2 overexpression was associated with a better prognosis in breast cancer [15]. Another study demonstrated that the antitumorigenic effects of DBC2 in breast cancer were specifically dependent on promoting polyubiquitination-mediated proteasomal degradation of MSI2 in breast cancer cell lines [27]. These results indicate that MSI2 isoforms expression and function in breast cancer progression remain to be defined and require further investigation.

Thus, we sought to examine the expression and biological functions of these MSI2 isoforms in TNBC. According to our results, canonical MSI2a and truncated MSI2b were the two dominant isoforms in the TNBC tissues evaluated. MSI2 in the TNBC tissues was downregulated compared with that in ANTs and other breast cancer subtypes, and loss of MSI2a expression in TNBC was associated with poor patient survival. The publicly available TNBC RNA-Seq data also indicated downregulation of MSI2a expression in TNBC vs ANT samples. Moreover, MSI2a overexpression reduced the viability of MDA-MB-231 and MDA-MB-468 cells, whereas knockdown of MSI2 expression promoted the viability of Hs-578 t and BT20 cells. MSI2a expression suppressed TNBC cell migration and wound healing, but knockdown of MSI2 promoted Hs-578 t and BT20 cell migration and wound healing. Additionally, MSI2a knockdown promoted BT20 cell viability, migration and wound healing, although MSI2b knockdown showed no significant effect on TNBC cells. To support our current findings, we conducted in vivo experiments and revealed that MSI2a overexpression significantly reduced the size of TNBC cell xenografts and tumor cell metastasis to the lungs in nude mice; conversely, MSI2b showed no significant effect on the TNBC phenotype. Our ex vivo data, involving several cohorts and different gene expression levels, confirmed the favorable role of MSI2a in TNBC. Furthermore, our study showed that MSI2a overexpression significantly downregulated the expression levels of p-ERK1/2 and the mesenchymal markers SLUG N-cadherin and vimentin but upregulated those of the epithelial markers ZO-1, β-catenin, and E-cadherin; these findings are consistent with data on MSI2 regulation of EMT markers ^14^. Further studies are also needed to elucidate the exact functions of isoform c and isoform d. In addition, we found that the antitumor activity of MSI2a occurred through its binding to and upregulation of TP53INP1, which then suppressed ERK1/2 activation in TNBC cells. Collectively, MSI2a possessed antitumor activities in TNBC.

Overall, further studies are needed to identify whether the role of MSI2a is organ-site dependent. Indeed, previous studies have shown that RBPs, such as MSI2, possess the dual capacity to stimulate and repress the translation of specific mRNAs, depending on the cellular context [7, 28, 29]. In our study, we demonstrated that MSI2a was able to bind to and upregulate TP53INP1 and a panel of p53 signaling genes that can regulate p53 phosphorylation, including TP53INP1, ATM, EP300, HIPK2, and DNA-PKcs [30]. Thus, impaired expression of these molecules might be involved in breast cancer pathogenesis and be associated with the poor survival of breast cancer patients [31–34]. Indeed, impaired expression of these genes together might contribute to the poor prognosis of TNBC patients with low MSI2a expression. Moreover, according to molecular assays, the major components of TNBC are basal-like tumors and other tumor types. Approximately 70–75% of TNBCs are basal-like cancers, and this type of breast cancer is thought to originate from normal basal/myoepithelial cells of the breast, which is in contrast to luminal cancers that originate from a differentiated luminal precursor cell [35]. These results may partly explain the discrepancies in MSI2 expression and function between TNBC and luminal breast cancer or other types of cancer.

TP53INP1 expression is often silenced in breast cancer cells and other human cancers, demonstrating that TP53INP1 is an unfavorable prognostic marker [36–38]. Loss of TP53INP1 expression in breast cancer cells is due to oncogenic factors, such as miR-569, which suppress TP53INP1 expression through posttranscriptional mechanisms. In the current study, we provided compelling biological data and ex vivo evidence showing that TP53INP1 plays a tumor suppressive role in TNBC. Specifically, TP53INP1 expression in TNBC was downregulated compared with that in normal tissues and other breast cancer subtypes. Knockdown of TP53INP1 expression induced ERK1/2 activation and promoted TNBC metastasis, while TP53INP1 overexpression suppressed ERK1/2 activation and TNBC metastasis. In addition, TP53INP1 expression was positively correlated with MSI2 expression in TNBC. TP53INP1 overexpression reversed the promotion of TNBC cell invasion induced by MSI2a knockdown. Our present study identified TP53INP1 as a binding partner and downstream gene of the MSI2a protein and established that the MSI2a-induced prosurvival effect on TNBC was at least partially through the posttranscriptional regulation of TP53INP1 expression. Moreover, downregulated TP53INP1 promoted hepatocellular carcinoma cell metastasis through the p73/DUSP10 pathway and activation of ERK1/2 [18]. Thus, MSI2a/TP53INP1 may suppress ERK1/2 activation and TNBC metastasis partly through the p73/DUSP10 pathway, while further investigations are still needed to confirm this hypothesis.

The ERK1/2 signaling pathway is one of the most important pathways contributing to cell proliferation and EMT during tumor progression [39–41]. Our current data revealed that MSI2a overexpression inhibited ERK1/2 phosphorylation and EMT in TNBC cells in vitro. Treatment of TNBC cells with the ERK inhibitor U0126 attenuated MSI2a-knockdown-induced cell migration. Furthermore, knockdown of TP53INP1 expression partially antagonized the effects of MSI2a overexpression on ERK1/2 inhibition, whereas TP53INP1 overexpression altered the effects of MSI2a knockdown on ERK1/2 activation in TNBC cells. These data indicate that downregulation of MSI2a sustains ERK1/2 pathway activation by targeting TP53INP1 expression in TNBC cells. However, future studies are needed to investigate the underlying molecular mechanisms and to verify MSI2a as a therapeutic target for TNBC.

## Conclusions

In conclusion, we investigated the expression levels and phenotypic functions of two major alternatively spliced MSI2 isoforms: MSI2a and MSI2b. Our findings demonstrated the downregulated expression of MSI2a in the development and progression of TNBC, suggesting that MSI2a expression may be a favorable marker for TNBC prognosis. We also revealed that MSI2a is a negative regulator of TNBC metastasis and that loss of MSI2a expression in TNBC leads to aberrant ERK1/2 activation and TNBC metastasis through reduced TP53INP1 transcript stability (Fig. 8e). Thus, targeting this axis might be a useful strategy for treating TNBC.

## Supplementary information

**Additional file 1: Table S1.** Clinicopathological characteristics of TNBC patients. **Table S2.** Transcriptome microarray analysis of differentially expressed mRNAs between 25 TNBC tissues and 5 ANTs. **Table S3.** Clinicopathological characteristics of breast cancer patients with different MSI2a or TP53INP1 mRNA expression. **Table S4.** Cox regression analysis of MSI2a mRNA expression and Clinicopathological factors predicting DFS and OS of breast cancer patients. **Table S5.** Correlation of MSI2a protein expression with Clinicopathological parameters of breast cancer. **Table S6.** Univariate and multivariate analysis of disease free survival and overall survival in breast cancer. **Table S7.** Correlation of MSI2a or TP53INP1 protein expression with Clinicopathological parameters of TNBC patients. **Table S8.** Univariate and multivariate analysis of disease-free survival and overall survival in TNBC. **Table S9.** Hs-578T RIP-sequencing. **Table S10.** Hs-578T  and MDA-MB-231 RNA-sequencing.

**Additional file 2: Figure S1.** MSI2 expression in breast cancer. **a** The CRN web portal (http://syslab4.nchu.edu.tw/) was used to interrogate GSE58135 datasets.MSI2–001(MSI2a) demonstrated downregulated in TNBC primary tumors compared to that in uninvolved breast tissue samples that were adjacent to TNBC primary tumors. **b** TCGA dataset. Levels of MSI2 mRNA across different breast cancer types in 737 breast tumors from the TCGA breast RNA-seq cohort(tcga-data.nci.nih.gov). **c** Transcripts abundance of MSI2 isoforms a-d between 25 TNBC tissues and 5 adjacent normal tissues (ANTs) of the RNAseq data. **d** qRT-PCR. MSI2a and MSI2b mRNA expression levels in 27 pairs of TNBC and normal tissues. **e** Kaplan–Meier survival curves comparing overall survival and disease-free survival of breast cancer patients with low vs. high MSI2a mRNA level. **f** qRT-PCR. MSI2b mRNA expression levels across different breast cancer types. **g** Receiver operating characteristic (ROC) curves of disease-free survival and overall survival showing the area under the ROC (AUROC) of MSI2b expression. **h** Kaplan–Meier survival curves comparing overall survival and disease-free survival of breast cancer patients with low vs. high MSI2a protein level. *p < 0.05, **p < 0.01, ***p < 0.001. **Figure S2.** MSI2b knockdown showed no significant effect on TNBC cell growth and migration in vitro. **a** qRT-PCR assays. The efficiency of MSI2b knockdown in HS-578T cells. **b** CCK-8 assay for cell viability. Cell proliferation was assessed after MSI2b knockdown inHs-578T cells. **c** Wound healing and transwell migration assay. Images showing the migration capacity in MSI2b-knocked down Hs-578T cells. **d** Western blot assays of SLUG, ZO-1, vimentin, E-cadherin, N-cadherin, β-catenin, p-ERK1/2, and ERK1/2 expression in MDA-MB-468 and BT20 cells. **e** Tumorigenicity assay of MSI2a-overexpressing MDA-MB-231 cells (2 × 10^6^) after subcutaneous injection in the flanks of nude mice (*n* = 5). The mice and tumors are shown. The quantification of tumor volume is shown below. Scale bar, 1 cm. **Figure S3.** Association of TP53INP1 expression with MSI2 expression. **a** Pathway analysis of enriched genes (log2 (fold change) below - 0.58 in RIP-seq) in HS-578T cells. The top three most significant pathways with enrichment scores are shown. **b** Expression of MSI2a and TP53INP1 mRNA was positively correlated in breast cancer. MSI2 and TP53INP1 mRNA expression levels were correlated using two published gene expression databases comprising 1215 breast tumors (**c**) from the TCGA breast RNA-seq cohort (tcga-data.nci.nih.gov) and 59 breast cell lines (**d**) from the Cancer Cell Line Encyclopedia breast cancer lines RNA-seq cohort. Statistical significance was determined using Pearson’s correlation. **e** Association of MSI2 and TP53INP1 mRNA expression in 166 TNBC tissues from the GSE76250 dataset. Statistical significance was determined using Pearson’s correlation.**Figure S4.** TP53INP1 inhibition of TNBC cell migration in vitro. **a** Transwell migration assay. Representative images and quantification showing the migration ability of MDA-MB-231 cells after TP53INP1 overexpression and of Hs-578 t cells after TP53INP1 knockdown. Scale bar, 100 μm. **b** Wound-healing assays. Representative images and quantification of the wound-healing assay results showing the wound-healing ability of MDA-MB-231 cells after TP53INP1 overexpression and of Hs-578T cells after TP53INP1 knockdown. Scale bar, 200 μm. **c** Western blot. TP53INP1-overexpressed MDA-MB-231 cells and TP53INP1-knocked down Hs-578T cells were analyzed by using western blot with the anti-TP53INP1, anti-P73, anti-DUSP10, anti-ERK, and anti-p-ERK antibodies, respectively. Vinculin was used as a control. *p < 0.05, **p < 0.01, ***p < 0.001. **Figure S5.** U0126 impairment of TNBC cell growth and migration induced by MSI2 silencing. a Wound-healing assay. Cells were treated with 10 μM U0126 or DMSO for 24 h and subjected to the wound-healing assay. U0126 attenuated the effect of MSI2a silencing on the scratch-closure rate of Hs-578T and BT20 cells. **b** Transwell tumor cell migration assay. Cells were treated with 10 μM U0126 or DMSO for 24 h and subjected to a Transwell assay. U0126 attenuated the effect of MSI2a silencing on the migration abilities of Hs-578T and BT20 cells. *p < 0.05. **Figure S6.** Association of TP53INP1 downregulation in human TNBC tissues with a poor TNBC prognosis. **a** Kaplan–Meier survival curves comparing overall survival and disease-free survival in TNBC patients with low vs. high TP53INP1 protein levels. **b** TCGA dataset. The level of TP53INP1 mRNA was analyzed using qRT-PCR in 27 pairs of TNBC tissues. **c** The levels of TP53INP1 mRNA across different breast cancer types were analyzed in 737 breast tumors from the TCGA breast RNA-seq cohort (tcga-data.nci.nih.gov) using qRT-PCR. **d** Kaplan–Meier survival curves comparing overall survival and disease-free survival in breast cancer patients with low vs. high TP53INP1 mRNA levels. *p < 0.05, **p < 0.01, and ***p < 0.001.

## Data Availability

All data in this study are included in this publication and related data files.

## Abbreviations

ANTs: Adjacent normal breast tissues
CCK-8: Cell counting kit-8
DFS: Disease-free survival
DMSO: Dimethyl sulfoxide
ERK1/2: Extracellular signal-regulated kinase 1/2
HR: Hazard ratio
MSI2: Musashi 2
OS: Overall survival
PCR: Polymerase chain reaction
qRT-PCR: Quantitative reverse transcription-PCR
RBP: RNA-binding protein
RIP: RNA immunoprecipitation
TCGA: The Cancer Genome Atlas
TNBC: Triple-negative breast cancer
TP53INP1: Tumor protein 53-induced nuclear protein 1
3′-UTR: 3′-untranslated region
