# Supplementary material for: RNA-binding protein MSI2 isoforms expression and regulation in progression of triple-negative breast cancer
Source: J Exp Clin Cancer Res. 2020 May 24;39:92. doi: 10.1186/s13046-020-01587-x (PMC7245804; doi:10.1186/s13046-020-01587-x)
Supplement: Supplementary file 1 — Additional file 1: Table S1. Clinicopathological characteristics of TNBC patients. Table S2. Transcriptome microarray analysis of differentially expressed mRNAs between 25 TNBC tissues and 5 ANTs. Table S3. Clinicopathological characteristics of breast cancer patients with different MSI2a or TP53INP1 mRNA expression. Table S4. Cox regression analysis of MSI2a mRNA expression and Clinicopathological factors predicting DFS and OS of breast cancer patients. Table S5. Correlation of MSI2a protein expression with Clinicopathological parameters of breast cancer. Table S6. Univariate and multivariate analysis of disease free survival and overall survival in breast cancer. Table S7. Correlation of MSI2a or TP53INP1 protein expression with Clinicopathological parameters of TNBC patients. Table S8. Univariate and multivariate analysis of disease-free survival and overall survival in TNBC. Table S9. Hs-578T RIP-sequencing. Table S10. Hs-578T and MDA-MB-231 RNA-sequencing. [file 13046_2020_1587_MOESM1_ESM.zip › Supplementary tables.docx]

Table S1. Clinicopathological characteristics of TNBC patients

| Variable | Overall (N=25) | | Overall (N=27) | |
| --- | --- | --- | --- | --- |
|  |  |  |  |  |
|  | N | % | N | % |
| Age, years |  |  |  |  |
| ≤50 | 9 | 36.00 | 10 | 37.03 |
| >50 | 16 | 64.00 | 17 | 62.96 |
| Tumor size, cm |  |  |  |  |
| <2 | 6 | 24.00 | 6 | 22.22 |
| 2≤T<5 | 18 | 72.00 | 19 | 70.37 |
| ≥5 | 1 | 4.00 | 2 | 7.41 |
| Histological grade |  |  |  |  |
| I/II | 5 | 20.00 | 9 | 33.33 |
| III | 20 | 80.00 | 18 | 66.67 |
| Node status |  |  |  |  |
| pN0 (none) | 17 | 68.00 | 18 | 66.67 |
| pN1 (1-3 LNs) | 4 | 16.00 | 3 | 11.11 |
| pN2 (4-9 LNs) | 3 | 12.00 | 4 | 14.81 |
| pN3 (≥10 LNs) | 1 | 4.00 | 2 | 7.41 |

Table S2. Transcriptome microarray analysis of differentially expressed mRNAs between 25 TNBC tissues and 5 ANTs.

Table S3. Clinicopathological characteristics of breast cancer patients with different MSI2a or TP53INP1 mRNA expression

| Variable | Overall (N=129) | | MSI2a | | | | | TP53INP1 | | | | |
| --- | --- | --- | --- | --- | --- | --- | --- | --- | --- | --- | --- | --- |
|  |  |  | Low expression (N=65) | | High expression (N=64) | |  | Low expression (N=55) | | High expression (N=74) | |  |
|  | N | % | N | % | N | % | *P* | N | % | N | % | *P* |
| Age, years |  |  |  |  |  |  | 0.253 |  |  |  |  | 0.386 |
| ≤50 | 62 | 48.06 | 28 | 43.08 | 34 | 53.13 |  | 24 | 43.64 | 38 | 51.35 |  |
| >50 | 67 | 51.94 | 37 | 56.92 | 30 | 46.88 |  | 31 | 56.36 | 36 | 48.65 |  |
| Tumor size, cm |  |  |  |  |  |  | 0.866 |  |  |  |  | 0.906 |
| <2 | 21 | 16.28 | 11 | 16.92 | 10 | 15.63 |  | 9 | 16.36 | 12 | 16.22 |  |
| 2≤T<5 | 98 | 75.97 | 48 | 73.85 | 50 | 78.13 |  | 41 | 74.55 | 57 | 77.03 |  |
| ≥5 | 10 | 7.75 | 6 | 9.23 | 4 | 6.25 |  | 5 | 9.09 | 5 | 6.76 |  |
| Histological grade |  |  |  |  |  |  | ***0.042*** |  |  |  |  | ***0.002*** |
| I/II | 67 | 51.94 | 28 | 43.08 | 39 | 60.94 |  | 20 | 36.36 | 47 | 63.51 |  |
| III | 62 | 48.06 | 37 | 56.92 | 25 | 39.06 |  | 35 | 63.64 | 27 | 36.49 |  |
| Node status |  |  |  |  |  |  | 0.269 |  |  |  |  | 0.783 |
| pN0 (none) | 60 | 46.51 | 28 | 43.08 | 32 | 50.00 |  | 24 | 43.64 | 36 | 48.65 |  |
| pN1 (1-3 LNs) | 33 | 25.58 | 21 | 32.31 | 12 | 18.75 |  | 16 | 29.09 | 17 | 22.97 |  |
| pN2 (4-9 LNs) | 12 | 9.30 | 5 | 7.69 | 7 | 10.94 |  | 4 | 7.27 | 8 | 10.81 |  |
| pN3 (≥10 LNs) | 18 | 13.95 | 7 | 10.77 | 11 | 17.19 |  | 7 | 12.73 | 11 | 14.86 |  |
| pNX* | 5 | 3.88 | 4 | 6.15 | 1 | 1.56 |  | 4 | 7.27 | 1 | 1.35 |  |
| Molecular subtype |  |  |  |  |  |  | 0.367 |  |  |  |  | 0.577 |
| Luminal A | 41 | 31.78 | 19 | 29.23 | 22 | 34.38 |  | 18 | 32.73 | 23 | 31.08 |  |
| Luminal B | 36 | 27.91 | 16 | 24.62 | 20 | 31.25 |  | 13 | 23.64 | 23 | 31.08 |  |
| Her2 subtype | 19 | 14.73 | 9 | 13.85 | 10 | 15.63 |  | 7 | 12.73 | 12 | 16.22 |  |
| TNBC | 33 | 25.58 | 21 | 32.31 | 12 | 18.75 |  | 17 | 30.91 | 16 | 21.62 |  |
| Local recurrence |  |  |  |  |  |  | 0.404 |  |  |  |  | 0.082 |
| absence | 26 | 20.16 | 15 | 23.08 | 11 | 17.19 |  | 15 | 27.27 | 11 | 14.86 |  |
| presence | 103 | 79.84 | 50 | 76.92 | 53 | 82.81 |  | 40 | 72.73 | 63 | 85.14 |  |
| Distant Metastasis |  |  |  |  |  |  | 0.832 |  |  |  |  | 0.34 |
| absence | 19 | 14.73 | 10 | 15.38 | 9 | 14.06 |  | 10 | 18.18 | 9 | 12.16 |  |
| presence | 110 | 85.27 | 55 | 84.62 | 55 | 85.94 |  | 45 | 81.82 | 65 | 87.84 |  |

Table S4. Cox regression analysis of MSI2a mRNA expression and Clinicopathological factors predicting DFS and OS of breast cancer patients

| Variable | DFS | | | | | | OS | | | | | |
| --- | --- | --- | --- | --- | --- | --- | --- | --- | --- | --- | --- | --- |
|  | Univariate analysis | | | Multivariate analysis | | | Univariate analysis | | | Multivariate analysis | | |
|  | HR | 95% CI | *P* | HR | 95% CI | *P* | HR | 95% CI | *P* | HR | 95% CI | *P* |
| MSI2a | 0.694 | 0.326-1.479 | 0.343 |  |  |  | 0.21 | 0.073-0.602 | ***0.007*** | 0.464 | 0.118-1.817 | 0.27 |
| TP53INP1 | 0.546 | 0.297-1.003 | 0.051 |  |  |  | 0.232 | 0.094-0.574 | ***0.002*** | 0.263 | 0.106-0.650 | ***0.004*** |
| Age | 1.31 | 0.670-2.560 | 0.43 |  |  |  | 1.945 | 0.721-5.245 | 0.189 |  |  |  |
| Tumor size | 1.415 | 0.783-2.556 | 0.25 |  |  |  | 2.077 | 0.896-4.819 | 0.089 |  |  |  |
| Histological grade | 1.935 | 1.029-3.638 | ***0.04*** | 1.659 | 0.866-3.179 | 0.127 | 2.388 | 0.981-5.812 | 0.055 |  |  |  |
| Node status | 1.098 | 0.841-1.433 | 0.494 |  |  |  | 1.018 | 0.699-1.483 | 0.924 |  |  |  |
| Molecular subtype | 1.369 | 1.048-1.790 | ***0.021*** | 1.299 | 0.985-1.713 | 0.064 | 1.832 | 1.209-2.775 | ***0.004*** | 1.694 | 1.132-2.535 | ***0.01*** |

Table S5. Correlation of MSI2a protein expression with Clinicopathological parameters of breast cancer

| Variable |  |  | Overall (N=388) | | MSI2a | | | | |
| --- | --- | --- | --- | --- | --- | --- | --- | --- | --- |
|  |  |  |  |  | Low expression (N=246) | | High expression (N=142) | |  |
|  |  |  | N | % | N | % | N | % | *P* |
| Age, years |  |  |  |  |  |  |  |  | 0.926 |
| ≤50 |  |  | 201 | 51.15 | 127 | 51.00 | 74 | 51.39 |  |
| >50 |  |  | 187 | 47.58 | 119 | 47.79 | 68 | 47.22 |  |
| Tumor size, cm |  |  |  |  |  |  |  |  | 0.143 |
| <2 |  |  | 188 | 47.84 | 111 | 44.58 | 77 | 53.47 |  |
| 2≤T<5 |  |  | 182 | 46.31 | 121 | 48.59 | 61 | 42.36 |  |
| ≥5 |  |  | 18 | 4.58 | 14 | 5.62 | 4 | 2.78 |  |
| Histological grade |  |  |  |  |  |  |  |  | ***0.038*** |
| I/II |  |  | 285 | 72.52 | 172 | 69.08 | 113 | 78.47 |  |
| III |  |  | 103 | 26.21 | 74 | 29.72 | 29 | 20.14 |  |
| Node status |  |  |  |  |  |  |  |  | 0.583 |
| pN0 (none) |  |  | 214 | 54.45 | 132 | 53.01 | 82 | 56.94 |  |
| pN1 (1-3 LNs) |  |  | 97 | 24.68 | 62 | 24.90 | 35 | 24.31 |  |
| pN2 (4-9 LNs) |  |  | 38 | 9.67 | 22 | 8.84 | 16 | 11.11 |  |
| pN3 (≥10 LNs) |  |  | 33 | 8.40 | 24 | 9.64 | 9 | 6.25 |  |
| pNX |  |  | 6 | 1.53 | 6 | 2.41 | 0 | 0.00 |  |
| Molecular subtype |  |  |  |  |  |  |  |  | ***＜0.001*** |
| Luminal A |  |  | 92 | 23.41 | 46 | 18.47 | 46 | 31.94 |  |
| Luminal B |  |  | 90 | 22.90 | 51 | 20.48 | 39 | 27.08 |  |
| Her2 subtype |  |  | 81 | 20.61 | 50 | 20.08 | 31 | 21.53 |  |
| TNBC |  |  | 125 | 31.81 | 99 | 39.76 | 26 | 18.06 |  |
| Local recurrence |  |  |  |  |  |  |  |  | ***＜0.001*** |
| absence |  |  | 358 | 91.09 | 218 | 87.55 | 140 | 97.22 |  |
| presence |  |  | 30 | 7.63 | 28 | 11.24 | 2 | 1.39 |  |
| Distant Metastasis |  |  |  |  |  |  |  |  | ***0.001*** |
| absence |  |  | 324 | 82.44 | 194 | 77.91 | 130 | 90.28 |  |
| presence |  |  | 64 | 16.28 | 52 | 20.88 | 12 | 8.33 |  |

Table S6. Univariate and multivariate analysis of disease free survival and overall survival in breast cancer

| Variable | DFS | | | | | | OS | | | | | |
| --- | --- | --- | --- | --- | --- | --- | --- | --- | --- | --- | --- | --- |
|  | Univariate analysis | | | Multivariate analysis | | | Univariate analysis | | | Multivariate analysis | | |
|  | HR | 95% CI | *P* | HR | 95% CI | *P* | HR | 95% CI | *P* | HR | 95% CI | *P* |
| MSI2a | 0.326 | 0.208-0.513 | ***＜0.001*** | 0.314 | 0.176-0.560 | ***＜0.001*** | 0.195 | 0.099-0.381 | ***＜0.001*** | 0.207 | 0.073-0.588 | ***0.003*** |
| Age | 0.927 | 0.597-1.438 | 0.734 |  |  |  | 1.469 | 0.766-2.815 | 0.247 |  |  |  |
| Tumor size | 1.223 | 0.838-1.786 | 0.296 |  |  |  | 1.74 | 1.005-3.013 | ***0.048*** | 1.522 | 0.860-2.696 | 0.15 |
| Histological grade | 1.3 | 0.834-2.026 | 0.247 |  |  |  | 1.808 | 0.951-3.439 | 0.071 |  |  |  |
| Node status | 1.827 | 1.173-2.845 | ***0.008*** | 1.856 | 1.191-2.891 | ***0.006*** | 3.083 | 1.549-6.139 | ***0.001*** | 2.922 | 1.458-5.854 | ***0.003*** |
| Molecular subtype | 1.116 | 0.926-1.345 | 0.248 |  |  |  | 1.261 | 0.951-1.674 | 0.107 |  |  |  |

Table S7. Correlation of MSI2a or TP53INP1 protein expression with Clinicopathological parameters of TNBC patients

| Variable | Overall (N=125) | | MSI2a | | | | | TP53INP1 | | | | |
| --- | --- | --- | --- | --- | --- | --- | --- | --- | --- | --- | --- | --- |
|  |  |  | Low expression (N=88 | | High expression (N=37 | |  | Low expression (N=100) | | High expression (N=25) | |  |
|  | N | % | N | % | N | % | *P* | N | % | N | % | *P* |
| Age, years |  |  |  |  |  |  | 0.49 |  |  |  |  | 0.371 |
| ≤50 | 60 | 46.51 | 44 | 48.35 | 16 | 42.11 |  | 46 | 46.00 | 14 | 56.00 |  |
| >50 | 65 | 50.39 | 44 | 48.35 | 21 | 55.26 |  | 54 | 54.00 | 11 | 44.00 |  |
| Tumor size, cm |  |  |  |  |  |  | 0.595 |  |  |  |  | 1 |
| <2 | 58 | 44.96 | 38 | 41.76 | 20 | 52.63 |  | 46 | 46.00 | 12 | 48.00 |  |
| 2≤T<5 | 61 | 47.29 | 45 | 49.45 | 16 | 42.11 |  | 49 | 49.00 | 12 | 48.00 |  |
| ≥5 | 6 | 4.65 | 5 | 5.49 | 1 | 2.63 |  | 5 | 5.00 | 1 | 4.00 |  |
| Histological grade |  |  |  |  |  |  | ***0.004*** |  |  |  |  | 0.075 |
| I/II | 81 | 62.79 | 50 | 54.95 | 31 | 81.58 |  | 61 | 61.00 | 20 | 80.00 |  |
| III | 44 | 34.11 | 38 | 41.76 | 6 | 15.79 |  | 39 | 39.00 | 5 | 20.00 |  |
| Node status |  |  |  |  |  |  | 0.603 |  |  |  |  | 0.63 |
| pN0 (none) | 80 | 62.02 | 56 | 61.54 | 24 | 63.16 |  | 66 | 66.00 | 14 | 56.00 |  |
| pN1 (1-3 LNs) | 24 | 18.60 | 18 | 19.78 | 6 | 15.79 |  | 18 | 18.00 | 6 | 24.00 |  |
| pN2 (4-9 LNs) | 11 | 8.53 | 6 | 6.59 | 5 | 13.16 |  | 9 | 9.00 | 2 | 8.00 |  |
| pN3 (≥10 LNs) | 10 | 7.75 | 8 | 8.79 | 2 | 5.26 |  | 7 | 7.00 | 3 | 12.00 |  |
| Local recurrence |  |  |  |  |  |  | 0.227 |  |  |  |  | 0.733 |
| absence | 110 | 85.27 | 75 | 82.42 | 35 | 92.11 |  | 87 | 87.00 | 23 | 92.00 |  |
| presence | 15 | 11.63 | 13 | 14.29 | 2 | 5.26 |  | 13 | 13.00 | 2 | 8.00 |  |
| Distant Metastasis |  |  |  |  |  |  | ***0.004*** |  |  |  |  | 0.074 |
| absence | 103 | 79.84 | 67 | 73.63 | 36 | 94.74 |  | 79 | 79.00 | 24 | 96.00 |  |
| presence | 22 | 17.05 | 21 | 23.08 | 1 | 2.63 |  | 21 | 21.00 | 1 | 4.00 |  |

Table S8. Univariate and multivariate analysis of disease-free survival and overall survival in TNBC

| Variable | DFS | | | | | | OS | | | | | |
| --- | --- | --- | --- | --- | --- | --- | --- | --- | --- | --- | --- | --- |
|  | Univariate analysis | | | Multivariate analysis | | | Univariate analysis | | | Multivariate analysis | | |
|  | HR | 95% CI | *P* | HR | 95% CI | *P* | HR | 95% CI | *P* | HR | 95% CI | *P* |
| MSI2a | 0.244 | 0.114-0.526 | ***0.012*** | 0.236 | 0.072-0.774 | ***0.017*** | 0.144 | 0.062-0.397 | ***0.028*** | 0.142 | 0.019-1.061 | ***0.057*** |
| TP53INP1 | 0,445 | 0.183-1.082 | 0.17 |  |  |  | 0.244 | 0.076-0.783 | 0.136 |  |  |  |
| Age | 1.159 | 0.576-2.332 | 0.678 |  |  |  | 2.055 | 0.780-5.411 | 0.145 |  |  |  |
| Tumor size | 1.361 | 0.764-2.424 | 0.296 |  |  |  | 1.598 | 0.759-3.365 | 0.218 |  |  |  |
| Histological grade | 0.937 | 0.476-1.846 | 0.851 |  |  |  | 1.677 | 0.670-4.197 | 0.27 |  |  |  |
| Node status | 2.122 | 1.060-4.248 | ***0.034*** | 2.118 | 1.058-4.241 | ***0.034*** | 5.651 | 2.033-15.709 | ***0.001*** | 5.475 | 1.967-15.238 | ***0.001*** |

Table S9. Hs-578t RIP-sequencing.

Table S10. Hs-578t and MDA-MB-231 RNA-sequencing.
