# Supplementary material for: RNA-binding protein MSI2 isoforms expression and regulation in progression of triple-negative breast cancer
Source: J Exp Clin Cancer Res. 2020 May 24;39:92. doi: 10.1186/s13046-020-01587-x (PMC7245804; doi:10.1186/s13046-020-01587-x)
Supplement: Supplementary file 2 — Additional file 2: Figure S1. MSI2 expression in breast cancer. a The CRN web portal (http://syslab4.nchu.edu.tw/) was used to interrogate GSE58135 datasets.MSI2–001(MSI2a) demonstrated downregulated in TNBC primary tumors compared to that in uninvolved breast tissue samples that were adjacent to TNBC primary tumors. b TCGA dataset. Levels of MSI2 mRNA across different breast cancer types in 737 breast tumors from the TCGA breast RNA-seq cohort(tcga-data.nci.nih.gov). c Transcripts abundance of MSI2 isoforms a-d between 25 TNBC tissues and 5 adjacent normal tissues (ANTs) of the RNAseq data. d qRT-PCR. MSI2a and MSI2b mRNA expression levels in 27 pairs of TNBC and normal tissues. e Kaplan–Meier survival curves comparing overall survival and disease-free survival of breast cancer patients with low vs. high MSI2a mRNA level. f qRT-PCR. MSI2b mRNA expression levels across different breast cancer types. g Receiver operating characteristic (ROC) curves of disease-free survival and overall survival showing the area under the ROC (AUROC) of MSI2b expression. h Kaplan–Meier survival curves comparing overall survival and disease-free survival of breast cancer patients with low vs. high MSI2a protein level. *p < 0.05, **p < 0.01, ***p < 0.001. Figure S2. MSI2b knockdown showed no significant effect on TNBC cell growth and migration in vitro. a qRT-PCR assays. The efficiency of MSI2b knockdown in HS-578T cells. b CCK-8 assay for cell viability. Cell proliferation was assessed after MSI2b knockdown inHs-578T cells. c Wound healing and transwell migration assay. Images showing the migration capacity in MSI2b-knocked down Hs-578T cells. d Western blot assays of SLUG, ZO-1, vimentin, E-cadherin, N-cadherin, β-catenin, p-ERK1/2, and ERK1/2 expression in MDA-MB-468 and BT20 cells. e Tumorigenicity assay of MSI2a-overexpressing MDA-MB-231 cells (2 × 10^6^) after subcutaneous injection in the flanks of nude mice (n = 5). The mice and tumors are shown. The quantification of tumor volume [file 13046_2020_1587_MOESM2_ESM.docx]

**Figure S1. MSI2 expression in breast cancer. A** The CRN web portal (http://syslab4.nchu.edu.tw/) was used to interrogate GSE58135 datasets.MSI2-001(MSI2a) demonstrated downregulated in TNBC primary tumors compared to that in uninvolved breast tissue samples that were adjacent to TNBC primary tumors. **B** TCGA dataset. Levels of MSI2 mRNA across different breast cancer types in 737 breast tumors from the TCGA breast RNA-seq cohort(tcga-data.nci.nih.gov). **C** Transcripts abundance of MSI2 isoforms a-d between 25 TNBC tissues and 5 adjacent normal tissues (ANTs) of the RNAseq data. **D** qRT-PCR. MSI2a and MSI2b mRNA expression levels in 27 pairs of TNBC and normal tissues. **E** Kaplan–Meier survival curves comparing overall survival and disease-free survival of breast cancer patients with low vs. high MSI2a mRNA level. **F** qRT-PCR. MSI2b mRNA expression levels across different breast cancer types. **G** Receiver operating characteristic (ROC) curves of disease-free survival and overall survival showing the area under the ROC (AUROC) of MSI2b expression. **H** Kaplan–Meier survival curves comparing overall survival and disease-free survival of breast cancer patients with low vs. high MSI2a protein level. *p<0.05, **p<0.01, ***p<0.001.

**Figure S2. MSI2b knockdown showed no significant effect on TNBC cell growth and migration in vitro. A** qRT-PCR assays. The efficiency of MSI2b knockdown in HS-578t cells. **B** CCK-8 assay for cell viability. Cell proliferation was assessed after MSI2b knockdown in HS-578t cells. **c** Wound healing and transwell migration assay. Images showing the migration capacity in MSI2b-knocked down HS-578t cells. **d** Western blot assays of SLUG, ZO-1, vimentin, E-cadherin, N-cadherin, β-catenin, p-ERK1/2, and ERK1/2 expression in MDA-MB-231 and Hs-578t cells. **e** Tumorigenicity assay of MSI2a-overexpressing MDA-MB-231 cells (2 × 10^6^) after subcutaneous injection in the flanks of nude mice (n = 5). The mice and tumors are shown. The quantification of tumor volume is shown below. Scale bar, 1 cm.

**Figure S3. Association of TP53INP1 expression with MSI2 expression.** **A** Pathway analysis of enriched genes (log2 (fold change) below - 0.58 in RIP-seq) in HS-578t cells. The top three most significant pathways with enrichment scores are shown. **B** Expression of MSI2a and TP53INP1 mRNA was positively correlated in breast cancer. MSI2 and TP53INP1 mRNA expression levels were correlated using two published gene expression databases comprising 1215 breast tumors (**C**) from the TCGA breast RNA-seq cohort (tcga-data.nci.nih.gov) and 59 breast cell lines (**D**) from the Cancer Cell Line Encyclopedia breast cancer lines RNA-seq cohort. Statistical significance was determined using Pearson’s correlation. **E** Association of MSI2 and TP53INP1 mRNA expression in 166 TNBC tissues from the GSE76250 dataset. Statistical significance was determined using Pearson’s correlation.

**Figure S4. TP53INP1 inhibition of TNBC cell migration in vitro. A** Transwell migration assay. Representative images and quantification showing the migration ability of MDA-MB-231 cells after TP53INP1 overexpression and of Hs-578t cells after TP53INP1 knockdown. Scale bar, 100 μm. **B** Wound-healing assays. Representative images and quantification of the wound-healing assay results showing the wound-healing ability of MDA-MB-231 cells after TP53INP1 overexpression and of HS-578t cells after TP53INP1 knockdown. Scale bar, 200 μm. **C** Western blot. TP53INP1-overexpressed MDA-MB-231 cells and TP53INP1-knocked down Hs-578t cells were analyzed by using western blot with the anti-TP53INP1, anti-P73, anti-DUSP10, anti-ERK, and anti-p-ERK antibodies, respectively. Vinculin was used as a control. *p<0.05, **p<0.01, ***p<0.001.

**Figure S5. U0126 impairment of TNBC cell growth and migration induced by MSI2 silencing.** **A** Wound-healing assay. Cells were treated with 10 µM U0126 or DMSO for 24 h and subjected to the wound-healing assay. U0126 attenuated the effect of MSI2a silencing on the scratch-closure rate of Hs-578t and BT20 cells. **B** Transwell tumor cell migration assay. Cells were treated with 10 µM U0126 or DMSO for 24 h and subjected to a Transwell assay. U0126 attenuated the effect of MSI2a silencing on the migration abilities of Hs-578t and BT20 cells. *p<0.05.

**Figure S6. Association of TP53INP1 downregulation in human TNBC tissues with a poor TNBC prognosis. A** Kaplan–Meier survival curves comparing overall survival and disease-free survival in TNBC patients with low vs. high TP53INP1 protein levels. **B** TCGA dataset. The level of TP53INP1 mRNA was analyzed using qRT-PCR in 27 pairs of TNBC tissues. **C** The levels of TP53INP1 mRNA across different breast cancer types were analyzed in 737 breast tumors from the TCGA breast RNA-seq cohort (tcga-data.nci.nih.gov) using qRT-PCR. **D** Kaplan–Meier survival curves comparing overall survival and disease-free survival in breast cancer patients with low vs. high TP53INP1 mRNA levels. *p<0.05, **p<0.01, and ***p<0.001.
